# Sacubitril/valsartan attenuates renal injury caused by cecal ligation and puncture via TLR4/NFκB/NLRP3 inhibition and reduced oxidative stress and apoptosis in rats

**DOI:** 10.1038/s41598-026-65493-6

**Published:** 2026-08-11

**Authors:** Reham H. Mohyeldin, Mahmoud Abdelnaser, Remon Roshdy Rofaeil, Mina Ezzat Attya, Niera M. S. Soliman, Michael Samuel Ayad, Ehab E. Sharata

**Affiliations:** 1 Department of Pharmacology & Toxicology, Faculty of Pharmacy, Deraya University, Minia, 61111 Egypt; 2 Department of Biochemistry, Faculty of Pharmacy, Deraya University, Minia, 61111 Egypt; 3 Department of Medical Pharmacology, Faculty of Medicine, Minia University, 61519 Minia, Egypt; 4 Department of Pathology, Faculty of Medicine, Minia University, Minia, 61519 Egypt; 5 Department of Medical Physiology, Faculty of Medicine, Minia University, Minia, 61519 Egypt; 6 Vascular Surgery Department, Faculty of Medicine, Minia University, Minia, 61519 Egypt

**Keywords:** CLP, Renal injury, Sacubitril, NFκB, Caspase-3, TLR4, NLRP3, Diseases, Medical research, Nephrology

## Abstract

Sepsis constitutes a life-threatening systemic inflammatory response triggered by infection. It remains among the leading causes of in-hospital mortality, claiming over 11 million lives annually. For the first time, this study aimed to evaluate the capacity of sacubitril/valsartan to protect against renal damage induced by cecal ligation and puncture (CLP) in an experimental rat model. The CLP procedure was employed to establish a rat model of sepsis. Renal oxidative stress markers including MDA, GSH, and SOD were quantified. ELISA was used to measure renal levels of IL-18, IL-1β, and caspase-3. Gene expression analysis of NFκB and TNF-α was conducted via qRT-PCR. Kidney function was evaluated by measuring serum urea and creatinine. NLRP3 protein expression was determined by western blotting, while TLR4 expression was assessed immunohistochemically. Renal tissue histology was also examined. CLP induction markedly elevated serum urea and creatinine, along with renal MDA, IL-18, IL-1β, TNF-α, NFκB, and caspase-3, and upregulated TLR4 and NLRP3 protein expression relative to the sham group, while GSH and SOD levels were notably diminished. Treatment with sacubitril/valsartan effectively ameliorated all of these biochemical and histopathological disturbances. Sacubitril/valsartan significantly attenuated CLP-induced renal injury through the suppression of inflammatory and apoptotic markers and the inhibition of the TLR4/NFκB/NLRP3/IL-1β signaling cascade.

**Supplementary Information:**

The online version contains supplementary material available at https://doi.org/10.1038/s41598-026-65493-6.

## Introduction

Sepsis is a critical medical condition defined by a systemic inflammatory response syndrome in which the entire body undergoes widespread inflammation. It arises in the context of a confirmed or suspected infection, often culminating in severe consequences such as multi-organ failure, and carries a notably high mortality rate that may reach 25%^1^. Sepsis constitutes a life-threatening systemic inflammatory response triggered by infection. It remains among the leading causes of in-hospital mortality, claiming over 11 million lives annually^2^.

Among the most frequent and life-threatening complications of sepsis in intensive care unit (ICU) patients is acute kidney injury (AKI), approximately six million cases of sepsis-associated AKI occur annually worldwide^3^, which accounts for more than 50% of ICU-associated AKI cases and is associated with substantially elevated mortality rates^4^. Accordingly, identifying novel and effective therapeutic strategies to promote kidney recovery following sepsis has become an urgent clinical imperative^5^. The pathophysiology underlying sepsis is extraordinarily complex. As the inflammatory response intensifies throughout the course of the disease, it drives the generation of oxidative stress and disturbs mitochondrial energy dynamics, ultimately resulting in multi-organ failure^6^.

Among the Toll-like receptor (TLR) family, TLR4 is prominently expressed on the surface membrane of renal tubular epithelial cells and occupies a pivotal position in the pathogenesis of sepsis-induced AKI^7^. The contribution of TLR4 to sepsis pathogenesis has been extensively established^8–10^; More recently, considerable scholarly attention has shifted toward the NLR family pyrin domain-containing 3 (NLRP3) as a member of the NLR receptor family. The ability of NLRP3 and related structures to assemble an inflammasome a multiprotein platform that converts pro-inflammatory pro-caspases into their active forms facilitates the production of critical pro-inflammatory cytokines^11,12^. As NF-κB becomes activated, it drives the transcription of NLRP3 inflammasome components together with pro-inflammatory cytokines such as TNF-α and IL-1β, thereby amplifying the systemic inflammatory response^13^. Sustained activation of this pathway disrupts intracellular and extracellular homeostasis, promoting immune cell infiltration, amplified oxidative stress, and eventual cellular apoptosis, all of which culminate in structural and functional renal damage.

Despite substantial research efforts dedicated to identifying effective anti-sepsis agents, the clinical management of sepsis-induced AKI remains far from satisfactory. Therefore, the development of innovative and efficacious therapeutic compounds is critically needed. This is especially pertinent given the well-supported hypothesis that inhibition of the TLR4/NF-κB-driven inflammatory response exerts protective effects against sepsis across multiple organs^14^.

LCZ696, known commercially as sacubitril/valsartan, is a dual-acting pharmaceutical compound comprising sacubitril a neprilysin inhibitor alongside valsartan, an angiotensin receptor blocker. This combination drug is approved for managing heart failure with reduced ejection fraction^15^. Its unique pharmacological mechanism involves simultaneous suppression of the renin-angiotensin-aldosterone system (RAAS) and inhibition of the neprilysin enzyme, thereby preventing the degradation of natriuretic peptides and augmenting their intracellular concentrations^16^. The RAAS plays a significant role in the control of sepsis and associated organ failure. Angiotensin II (Ang II) the principal active mediator of RAAS is a potent pro-inflammatory molecule whose primary receptor, the angiotensin II type 1 receptor (AT1R), is expressed across multiple tissues, including renal parenchyma and immune cells^17^.

It is noteworthy that studies examining NLRP3’s role in renal injury have demonstrated that Ang II-mediated activation of NF-κB and NLRP3 drives inflammation, proteinuria, and renal tubular cell pyroptosis, all of which contribute to kidney injury precipitated by diverse stimuli^18^. Furthermore, recent evidence has shown that atrial natriuretic peptide (ANP) and B-type natriuretic peptide (BNP) exert potent inhibitory effects on all pathways of NLRP3 inflammasome activation in human monocytic cells through the regulation of multiple molecular mechanisms^19^. Collectively, these observations illuminate the intricate relationship between the TLR4/NLRP3/NF-κB pathway and the crosstalk between RAAS activation and the natriuretic peptide system in the context of sepsis.

Encouragingly, prior research has demonstrated that LCZ696 is capable of suppressing the NF-κB/NLRP3 inflammasome, thereby attenuating inflammatory responses and conferring protection in a rat model of cyclophosphamide-induced premature ovarian failure. It has also demonstrated promise in mitigating early diabetic nephropathy and in ameliorating endothelial dysfunction through the TLR4/NF-κB signaling pathway^20–23^. Similarly, LCZ696 has been reported to effectively regulate inflammation and oxidative stress by elevating endogenous vasoactive peptide levels through neprilysin inhibition^24^.

Of particular concern is the markedly elevated mortality observed in patients presenting with concurrent cardiac dysfunction and sepsis, which may reach as high as 90%. These patients therefore require urgent and targeted therapeutic intervention. Managing heart failure alongside sepsis poses a formidable clinical challenge given the complex interplay between both conditions, as conventional heart failure treatments may not be suitable in the context of active sepsis and vice versa. Accordingly, in addition to its well-documented cardioprotective properties in heart failure patients, LCZ696 may represent a compelling therapeutic approach for alleviating sepsis-induced acute kidney injury^25^.

Therefore, the primary aim of the present study was to investigate the potential renoprotective benefits and underlying mechanisms of LCZ696 in relation to acute kidney injury, utilizing a well-established rat model of experimental sepsis induced by cecal ligation and puncture (CLP). This model is widely employed due to its close resemblance to the clinical course and hallmark features of human sepsis^26^. Briefly, CLP entails surgical ligation of the cecum followed by puncture, allowing controlled leakage of fecal material into the peritoneal cavity; this generates a polymicrobial peritonitis that progresses to systemic sepsis, reproducing the biphasic hemodynamic, immunological, and metabolic derangements characteristic of the clinical septic response, which is why this model remains the reference standard for sepsis research.

## Materials and methods

### Drugs and chemicals

LCZ696 was supplied by Novartis Pharma AG (Basel, Switzerland).

### CLP model of sepsis

Anesthesia was induced intraperitoneally using ketamine (100 mg/kg) and xylazine (10 mg/kg)^27^. Animals assigned to the sham group underwent laparotomy and bowel manipulation without CLP induction. Experimental sepsis was established using the CLP procedure as previously described^28^. Briefly, under anesthesia the cecum was exteriorized through a midline laparotomy, ligated distal to the ileocecal valve to preserve intestinal continuity, and punctured to allow extrusion of a small amount of fecal content, thereby standardizing the polymicrobial insult; the abdominal wall was then closed in layers and animals recovered with free access to food and water. Clinical signs consistent with sepsis (piloerection, lethargy, and reduced activity) were monitored qualitatively during the post-operative period; a formal quantitative severity score, such as serial core temperature measurement.

### Animals and experimental design

Adult male Wistar albino rats with body weights ranging from 180 to 210 g were obtained from the Animal House of Nahda University, Beni-Suef, Egypt. Only male rats were used in order to avoid the known immunomodulatory and anti-inflammatory influence of estrogen on the sepsis-associated inflammatory response, which is a recognized source of variability in CLP models; extending the present findings to female cohorts is an important direction for future work. Prior to experimental commencement, animals underwent a one-week acclimatization period under controlled laboratory conditions with unrestricted access to standard food pellets and water. Housing was maintained at a temperature of 25 ± 2 °C, a relative humidity of 45 ± 5%, and a 12-hour light/dark cycle^29^. Approval of our study was provided by the institutional Ethical Committee of Deraya Center for Scientific Research (Deraya University, Minia, Egypt) following NIH Guidelines (NIH Publications No. 8023, revised 1978) and complied with the ARRIVE guidelines (Approval Number: DCSR-03026-114).

**Rats were randomly assigned into 4 groups (n = 10/each group)**:

#### Sham group

rats received corn oil (2 ml/kg/day; p.o.) for 6 consecutive days, while laparotomy without CLP was performed on day 6^20,30^.

#### LCZ696 group

rats were orally administered LCZ696 dissolved in corn oil (30 mg/kg/day; p.o.) for 6 days^20,30^.

#### CLP group

rats were administered corn oil (2 ml/kg/day; p.o.) for 6 days, while CLP was carried out on the 6th day.

#### CLP+LCZ696 treated group

rats were given LCZ696 (30 mg/kg/day; p.o.) for 6 days, while CLP was carried out on the 6th day^20,30^.

### Blood and tissue sampling

On day 7, animals were deeply anesthetized with urethane (1.3 g/kg, i.p.)^30^. Blood was withdrawn from the abdominal aorta and centrifuged at 4000 × g for 15 min for serum biochemical analysis. Animals were euthanized via decapitation, and renal tissues were promptly harvested. Tissue and serum sampling was performed 24 h after CLP induction, a time point at which renal functional impairment and pro-inflammatory/oxidative injury are consistently reported to peak in this model, while remaining early enough to precede the substantial CLP-associated mortality that develops at later time points; this window therefore offers a reproducible balance between capturing maximal injury and preserving adequate group sizes for analysis. Kidney tissue was divided into four portions: the first two were immediately stored at − 80 °C for subsequent western blot and qRT-PCR analyses; the third portion was stored at − 80 °C for biochemical evaluation following homogenization in 10 mM potassium phosphate buffer (pH 7.4); and the fourth portion was fixed in 10% formalin for histopathological assessment.

### Biochemical assessment

#### Estimation of kidney function tests

In accordance with the manufacturer’s guidelines, serum urea was quantified using a commercial kit (Cat. No.: 1001331, SPINREACT, Ctra. Santa Coloma, Spain), and serum creatinine was measured using a dedicated assay kit (Cat. No.: 11734, Biosystems, Barcelona, Spain).

#### Oxidative stress markers determination

Renal reduced glutathione (GSH; Cat. No.: GR 25 11), superoxide dismutase (SOD; Cat. No.: SD 25 21), and malondialdehyde (MDA; Cat. No.: MD 25 29) were all determined using commercially available kits from Biodiagnostic, Giza, Egypt.

#### Estimation of renal inflammatory markers

Renal concentrations of IL-18 (Cat. No.: E-EL-R0567) and IL-1β (Cat. No.: E-EL-R0012) were measured using ELISA kits obtained from Elabscience (Houston, TX, USA), following the manufacturer’s recommended procedures.

#### Renal cleaved caspase-3 assessment

Renal cleaved caspase-3 was detected using an ELISA kit (MBS1605634, MyBioSource, USA) following the manufacturer’s specifications.

### Quantitative real-time polymerase chain reaction

Renal tissue samples (100 mg each) were ultrasonically homogenized in 1 mL of TRIzol reagent using a Branson Digital Sonifier ultrasonic cell homogenizer (SFX 550, Danbury, Connecticut, United States). Total RNA quantity was determined, and purity was assessed via the A260/A280 absorption ratio. Only RNA samples with purity values ≥ 1.7 were used for qRT-PCR analysis. Equal amounts of total RNA were reverse transcribed into complementary DNA using the Revert Aid First Strand cDNA Synthesis Kit (Cat no.: K1622, Thermofisher Scientific, United Kingdom). Real-time PCR was subsequently conducted using single-stranded cDNA templates. Primer sequences are detailed in Table 1. PCR amplification was carried out using Thermo Scientific Maxima SYBR Green qPCR Master Mix (2X) (Cat no.: K0251, Thermofisher Scientific, United Kingdom) on a StepOne Real-Time PCR Detection System (Ref no.: 4369074, Applied Biosystems, Singapore). mRNA expression levels were normalized to GAPDH as an internal reference gene, and quantification was performed using the comparative cycle threshold method^31^.

**Table 1:**
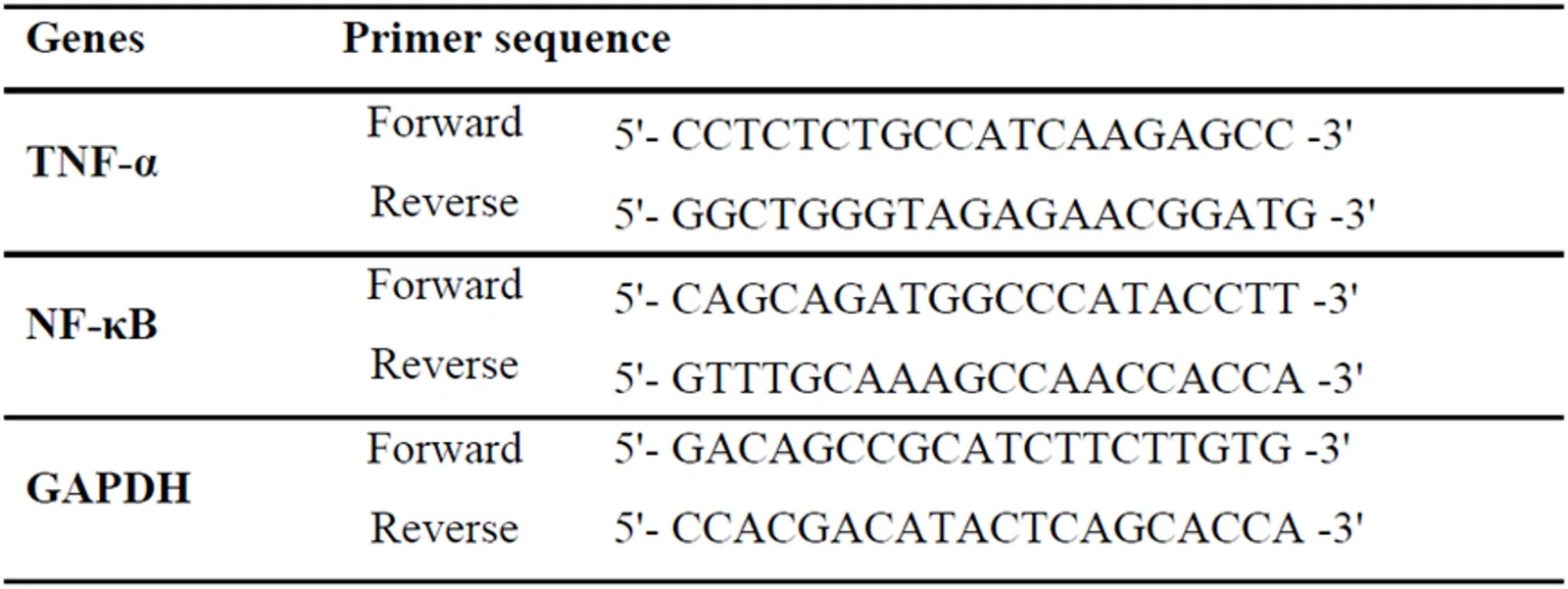
The primer sequences.

### Immunohistochemical staining

Paraffin-embedded renal tissue Sect. (5 μm thick) were deparaffinized and rehydrated for immunohistochemical analysis. Antigen retrieval was performed by immersing sections in a Tris–EDTA retrieval buffer (pH 9.0) and placing them in a water bath at 98 °C for 20 min to expose masked antigens. Endogenous peroxidase activity was subsequently quenched by applying a 3% hydrogen peroxide (H2O2) solution for 15 min. To minimize non-specific background staining, sections were blocked with 5% normal rabbit serum. Thereafter, sections were incubated with primary antibodies targeting TLR4 (Bioss Antibodies, China), followed by secondary antibody staining using a goat anti-rabbit biotinylated antibody for 20 min. A 20-minute incubation with pre-diluted streptavidin–horseradish peroxidase was then performed. Antibody binding was visualized using DAB as a chromogen, and counterstaining was carried out with Mayer’s hematoxylin. The degree of immunoreactivity in renal tissues was evaluated by analyzing the area of positive expression using ImageJ software^32^.

### Western blotting analysis

Renal homogenates containing 30 µg of total protein were denatured for 5 min in loading buffer containing 2-mercaptoethanol, resolved by 12% SDS-PAGE for 2 h, and subsequently transferred onto polyvinylidene fluoride (PVDF) membranes. Non-specific binding sites were blocked using a TBS-T solution containing 5% (w/v) non-fat dry milk and 0.05% Tween-20. Membranes were then incubated overnight at 4 °C with primary antibodies, including anti-NLRP3 (1:1000, ab263899, Abcam, UK) and β-actin (Santa Cruz Biotechnology, Santa Cruz, CA). Detection was achieved using a horseradish peroxidase-conjugated secondary polyclonal immunoglobulin (1:5000; Cell Signaling Technology Inc., MA, USA) diluted in blocking buffer. Protein bands were visualized by chemiluminescence, and densitometric quantification was performed using ImageJ software, with results expressed as fold changes relative to the sham group^33^. During the experimental procedure, membranes were cut prior to antibody hybridization to allow simultaneous incubation with different primary antibodies (targeting proteins of distinct molecular weights) and to conserve reagents. All replicates are included in the Supplementary Information.

### Histopathology

Kidneys from all animals were promptly fixed in 10% formalin, routinely processed, and stained with hematoxylin and eosin (H&E) according to standard histological procedures^34^. Slides were examined using an Olympus (U.TV0.5XC-3) light microscope fitted with a high-resolution digital camera. Assessment was performed in a blinded and randomized manner. Histopathological alterations in renal tissue were categorized as 0: none, 1: mild, 2: moderate, and 3: severe, depending on the degree of inflammatory cell infiltration as well as renal tissue degradation and necrosis^32^.

### Statistical analysis

All data are expressed as mean ± standard deviation (SD). Statistical comparisons were performed using one-way analysis of variance (ANOVA) followed by the Tukey-Kramer post-hoc test. A P-value of less than 0.05 was considered statistically significant. All analyses were conducted using GraphPad Prism^®^ Version 10.00 for Windows.

## Results

### LCZ696’s influence on kidney functions

**Fig. 1:**
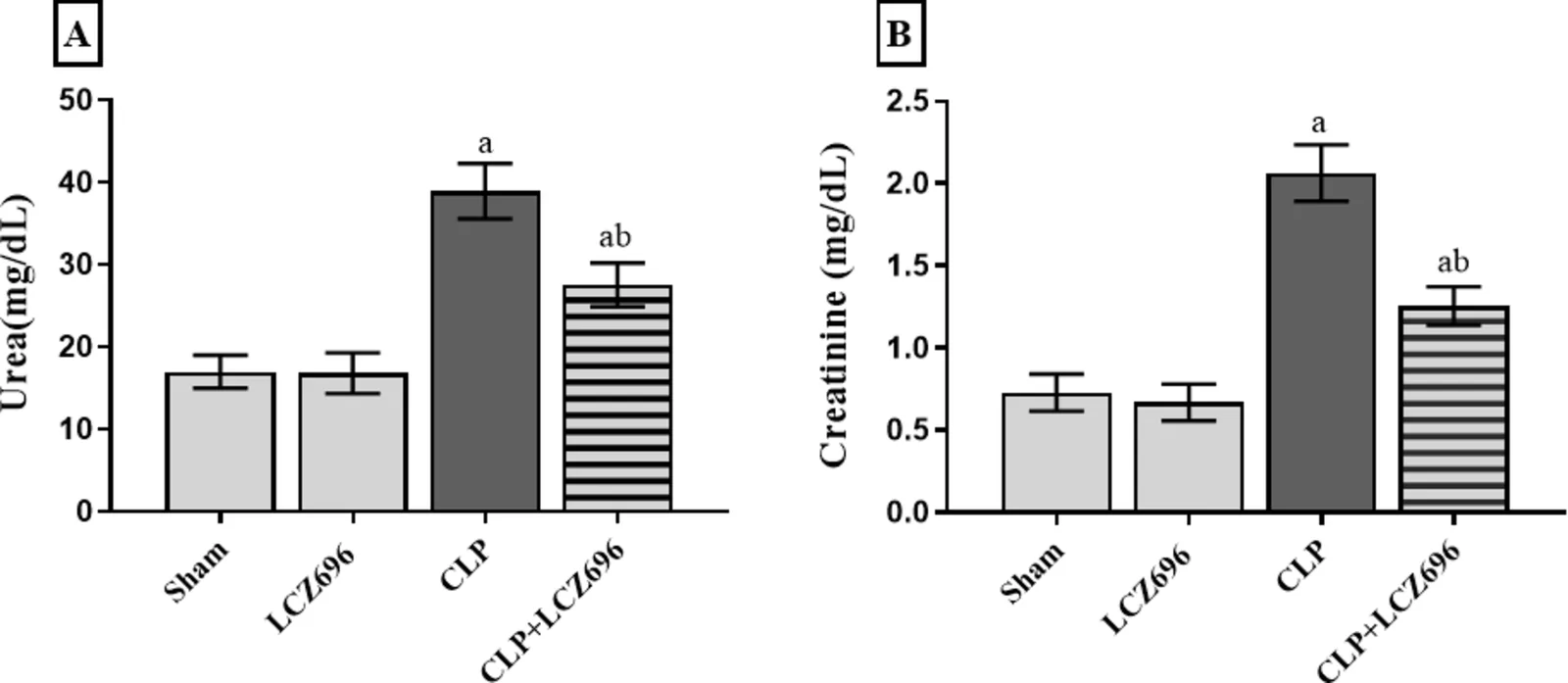
Serum concentrations of urea (**A**) and creatinine (**B**). Values are expressed as mean ± SD (*n* = 6). Statistical significance is set at *P* < 0.05, as determined by one-way ANOVA followed by the Tukey-Kramer post-hoc test. (a) significant difference relative to the sham group; (b) significant difference from the CLP group. CLP: cecal ligation-puncture.

Assessment of renal function markers revealed significant perturbations in the CLP-induced sepsis group. As shown in Fig. 1A–B, animals subjected to CLP exhibited a statistically significant elevation in serum urea (+ 129.3% versus sham, *P* < 0.0001) and creatinine (+ 183.3% versus sham, *P* < 0.0001) concentrations when compared to the sham-operated control group. The rise in both biomarkers was consistent with a severe impairment of glomerular filtration capacity, reflecting the acute kidney injury profile typically observed in this experimental sepsis model. Urea accumulation in the bloodstream points to disrupted nitrogen metabolism secondary to reduced renal clearance, while elevated creatinine signifies compromised tubular and glomerular function. Together, these observations confirm that CLP effectively induced clinically relevant renal dysfunction in the treated animals.

In marked contrast, pretreatment with LCZ696 prior to CLP induction resulted in a significant reduction in both serum urea (-29.2% versus CLP, *P* < 0.0001) and creatinine (-39.2% versus CLP, *P* < 0.0001) levels compared to the untreated CLP group. This normalization of renal function markers indicates that LCZ696 effectively preserved glomerular filtration function and mitigated the nephrotoxic burden imposed by systemic sepsis. **3.2. LCZ696’s impact on renal contents of MDA**,** GSH**,** and SOD**.

Analysis of renal tissue homogenates revealed that the CLP group displayed a statistically significant decline in the levels of the antioxidant markers GSH (-62.0% versus sham, *P* < 0.0001) and SOD (-38.9% versus sham, *P* < 0.0001) compared to the sham group. This depletion of both enzymatic (SOD) and non-enzymatic (GSH) antioxidants indicates a substantial compromise of the kidney’s intrinsic scavenging capacity. Their marked reduction in the CLP group confirms that sepsis triggers a state of profound renal oxidative stress.

**Fig. 2:**
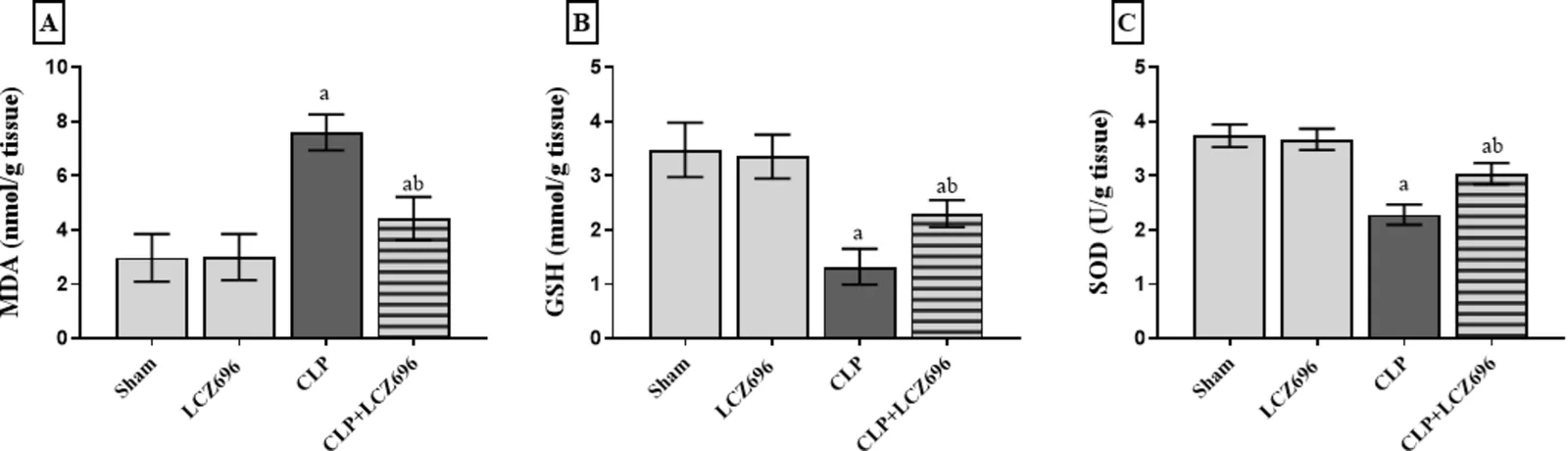
Renal tissue levels of MDA (**A**), GSH (**B**), and SOD (**C**). Data are expressed as mean ± SD (*n* = 6). Statistical significance is set at *P* < 0.05, as determined by one-way ANOVA followed by the Tukey-Kramer post-hoc test. (a) significant difference relative to the sham group; (b) significant difference from the CLP group. GSH: reduced glutathione; SOD: superoxide dismutase; MDA: malondialdehyde; CLP: cecal ligation-puncture.

Concurrently, renal MDA levels, a validated marker of lipid peroxidation and oxidative membrane damage, were significantly elevated in the CLP group relative to sham-operated animals (+ 155.4% versus sham, *P* < 0.0001). This increase in MDA reflects the excessive peroxidation of lipid membranes within renal cells under the oxidative conditions imposed by CLP-induced sepsis and is indicative of ongoing cellular injury within the renal tissue. Importantly, pretreatment with LCZ696 significantly reversed these oxidative imbalances. The CLP+LCZ696 group demonstrated a significant increase in both SOD (+ 33.1% versus CLP, *P* < 0.0001) and GSH (+ 74.1% versus CLP, *P* = 0.0014) alongside a significant reduction in MDA content (-41.9% versus CLP, *P* < 0.0001) compared to the CLP-only group. As illustrated in Fig. 2A-C.

### LCZ696’s impact on renal IL-1β, and IL-18

**Fig. 3:**
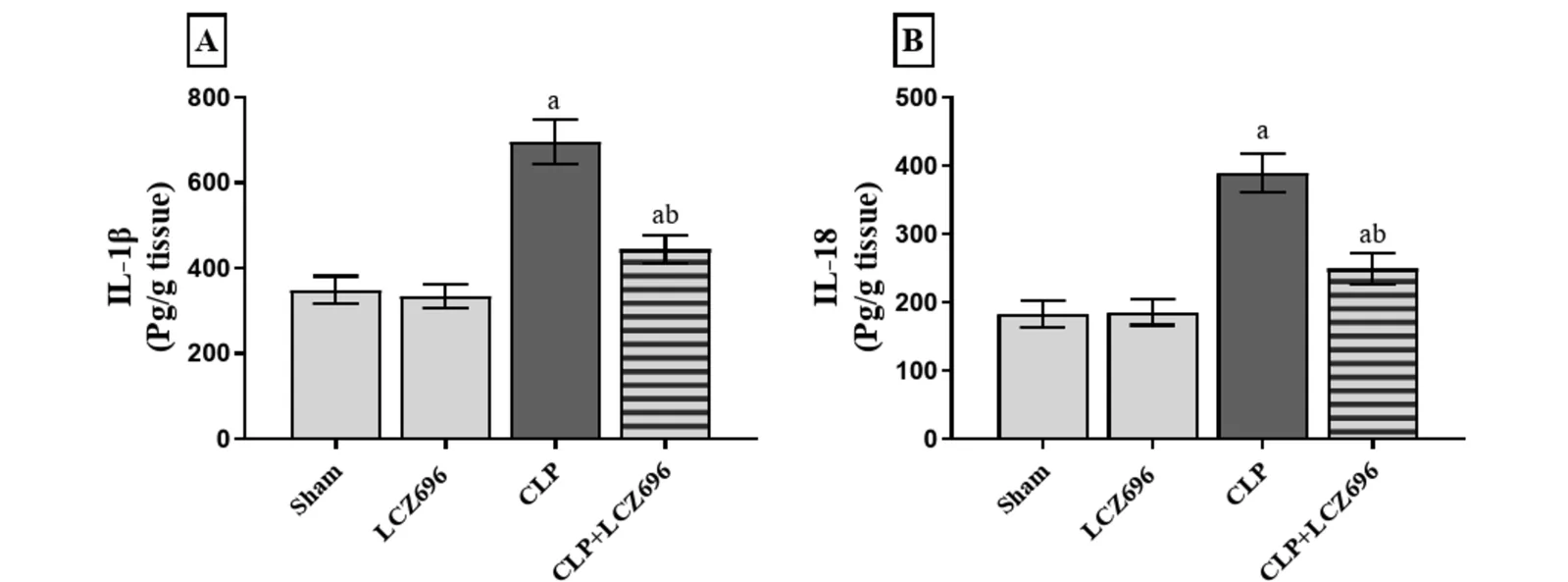
Renal tissue concentrations of the inflammatory markers IL-1β (**A**) and IL-18 (**B**). Values are expressed as mean ± SD (*n* = 6). Statistical significance is set at *P* < 0.05, as determined by one-way ANOVA followed by the Tukey-Kramer post-hoc test. (a) significant difference relative to the sham group; (b) significant difference relative to the CLP group. IL-18: interleukin-18; IL-1β: interleukin-1β; CLP: cecal ligation-puncture.

As shown in Fig. 3, the CLP group exhibited a significant increase in renal IL-1β (+ 99.3% versus sham, *P* < 0.0001) and IL-18 (+ 113.5% versus sham, *P* < 0.0001) protein concentrations compared to the sham group, reflecting robust inflammasome-mediated inflammatory signaling in the kidney. The substantial elevation of these cytokines in the CLP group is consistent with the intense inflammatory milieu induced by polymicrobial sepsis and aligns with the exacerbated renal tissue injury observed histopathologically. In marked contrast, pretreatment with LCZ696 led to a significant attenuation of both IL-1β (-36.2% versus CLP, *P* < 0.0001) and IL-18 (-36.0% versus CLP, *P* < 0.0001) levels in the CLP+LCZ696 group relative to the CLP group. The suppression of these key inflammasome-derived interleukins indicates that LCZ696 effectively interferes with the inflammatory signal transduction cascade in renal tissue, thereby curtailing cytokine storm and its deleterious downstream consequences on kidney structure and function.

### LCZ696’s influence on renal caspase 3

**Fig. 4:**
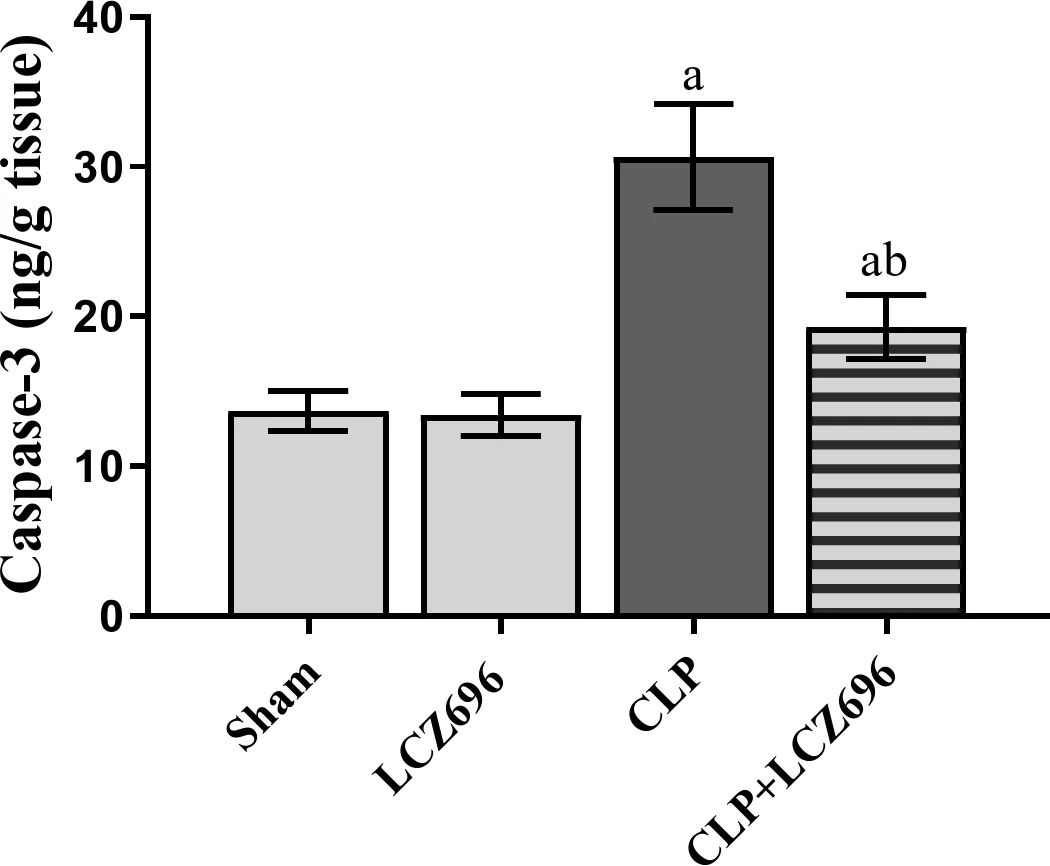
Renal tissue levels of cleaved caspase-3. Data are expressed as mean ± SD (*n* = 6). Statistical significance is set at *P* < 0.05, as determined by one-way ANOVA followed by the Tukey-Kramer post-hoc test. (**a**) significant difference relative to the sham group; (**b**) significant difference relative to the CLP group. CLP: cecal ligation-puncture.

Caspase-3 serves as a critical effector protease in the intrinsic apoptotic cascade, and its activation has been extensively documented in models of sepsis-induced acute kidney injury. As depicted in Fig. 4, renal caspase-3 levels were significantly elevated in CLP-induced rats relative to the sham-operated group (+ 124.0% versus sham, *P* < 0.0001). This marked upregulation of caspase-3 corroborates the presence of robust pro-apoptotic activity within renal tubular cells during experimental sepsis, indicating that CLP effectively triggered programmed cell death pathways in the kidney. The apoptotic loss of renal tubular cells under septic conditions directly contributes to structural disintegration and functional deterioration of the organ. In contrast, rats pretreated with LCZ696 demonstrated a statistically significant reduction in renal caspase-3 content compared to the CLP group (-37.1% versus CLP, *P* < 0.0001).

### LCZ696’s influence on NFκB and TNF-α genes

**Fig. 5:**
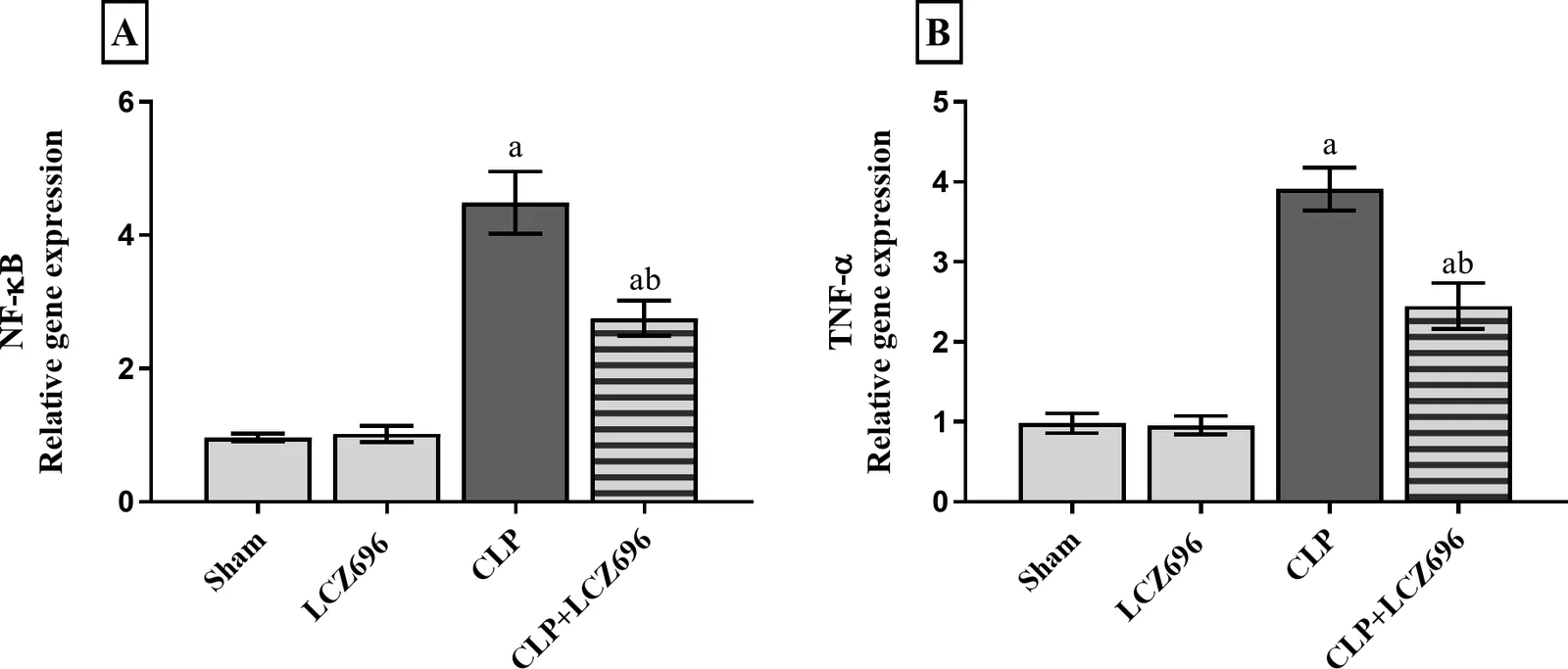
Renal mRNA expression levels of NFκ-B (**A**) and TNF-α (**B**), as determined by qRT-PCR. Gene expression was normalized to GAPDH and compared to the sham group. Data are expressed as mean ± SD (*n* = 6). Statistical significance is set at *P* < 0.05, as determined by one-way ANOVA followed by the Tukey-Kramer post-hoc test. (a) significant difference from the sham group; (b) significant difference from the CLP group. NFκ-B: nuclear factor-kappa B; TNF-α: tumor necrosis factor-α; CLP: cecal ligation-puncture.

Transcriptional regulation by NF-κB represents a central molecular event in the amplification of inflammatory gene expression during sepsis. As demonstrated in Fig. 5A–B, quantitative real-time PCR analysis revealed a significant upregulation of both NFκB (+ 366.1% versus sham, *P* < 0.0001) and TNF-α (+ 297.6% versus sham, *P* < 0.0001) mRNA levels in the renal tissues of CLP-treated rats compared to the sham group. This finding confirms that CLP-induced polymicrobial sepsis triggers robust transcriptional activation of key inflammatory mediators at the renal level, establishing a proinflammatory gene expression milieu that perpetuates tissue injury. TNF-α, as an early and potent proinflammatory cytokine, further amplifies NF-κB activation in a positive feedback loop, exacerbating the inflammatory cascade within the kidney. Remarkably, pretreatment with LCZ696 significantly suppressed both NFκB (-38.7% versus CLP, *P* = 0.0003) and TNF-α (-37.4% versus CLP, *P* = 0.0001) mRNA expression in renal tissues compared to the CLP group. The marked reduction in these pro-inflammatory gene transcripts following LCZ696 administration highlights the compound’s capacity to interfere with the upstream transcriptional drivers of sepsis-induced renal inflammation.

### LCZ696’s influence on TLR4 immune expression

**Fig. 6:**
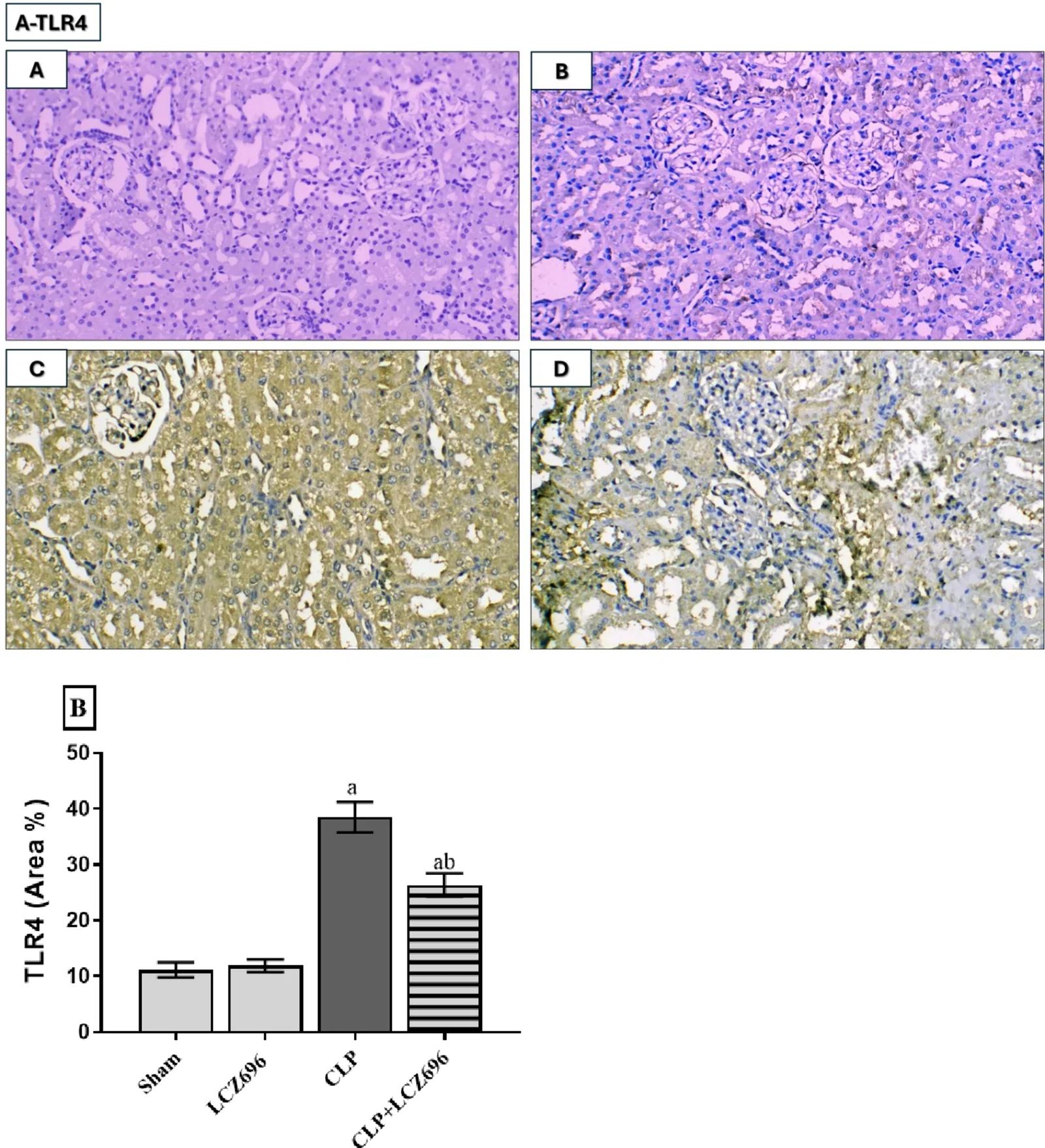
Photomicrographs demonstrating the impact of LCZ696 on the renal expression of TLR4 (**A**) from various animal groups; including control (A), LCZ696 (**B**), CLP (**C**), and CLP+LCZ696 (**D**) (x200). TLR4 positivity scoring (B). Data are expressed as mean ± SD (*n* = 3). Statistical significance is set at *P* < 0.05, as determined by one-way ANOVA followed by the Tukey-Kramer post-hoc test. (a) significant difference from the sham group; (b) significant difference from the CLP group. CLP: cecal ligation-puncture and TLR4: toll-like receptor 4.

Immunohistochemical analysis was employed to evaluate TLR4 protein expression within renal tissue sections, as illustrated in Fig. 6. The CLP group demonstrated a significantly elevated TLR4 immunostaining signal compared to the sham group (+ 246.5% versus sham, *P* < 0.0001), indicating pronounced upregulation of this innate immune pattern recognition receptor in renal tubular cells during experimental sepsis. The intensity and distribution of TLR4-positive staining in the CLP group underscored the central role of TLR4 in orchestrating the innate immune response within the septic kidney. Administration of LCZ696 prior to CLP induction resulted in a significant reduction in TLR4 immunoreactivity within renal tissue sections relative to the CLP group (-31.6% versus CLP, *P* = 0.0003). The capacity of LCZ696 to downregulate TLR4 protein expression at the cellular level suggests that modulation of the TLR4 innate immune signaling axis is a key mechanistic component of its anti-inflammatory activity within renal tissue during sepsis.

### LCZ696’s influence on NLRP3 protein expression

**Fig. 7:**
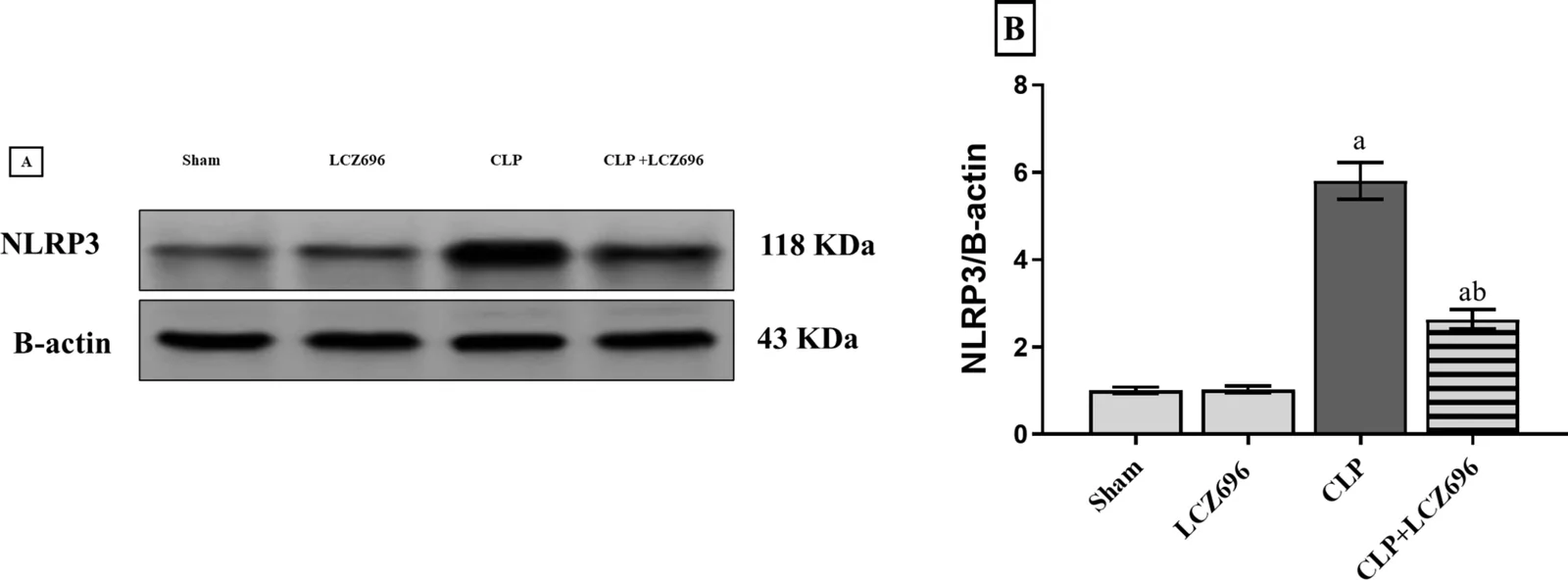
Representative western blot images (**A**) and quantified NLRP3 protein expression levels in renal tissue (**B**). Data are expressed as mean ± SD (*n* = 3). Statistical significance is set at *P* < 0.05, as determined by one-way ANOVA followed by the Tukey-Kramer post-hoc test. (a) significant difference from the sham group; (b) significant difference from the CLP group. NLRP3: NOD-like receptor family pyrin domain-containing 3; CLP: cecal ligation-puncture.

Western blot analysis was performed to quantify NLRP3 protein expression in renal tissue, as illustrated in Fig. 7A–B. The CLP-induced sepsis group exhibited a significant upregulation of NLRP3 protein levels compared to the sham group (+ 474.9% versus sham, *P* < 0.0001), confirming robust inflammasome-related signaling activation in the septic kidney. NLRP3 serves as the core scaffold protein of the NLRP3 inflammasome complex, which orchestrates the maturation and release of IL-1β and IL-18 and drives caspase-1-dependent and caspase-3-mediated cell death. The heightened NLRP3 expression observed in the CLP group underscores its pathological role as a major driver of the renal inflammatory injury cascade in this experimental model. In contrast, pretreatment with LCZ696 led to a statistically significant downregulation of NLRP3 protein expression in renal tissue compared to the CLP group (-54.6% versus CLP, *P* < 0.0001). These findings indicate that LCZ696 effectively suppresses inflammasome assembly and the associated signaling cascade within the septic kidney. By restraining NLRP3 protein upregulation, LCZ696 disrupts the inflammasome-driven amplification of cytokine production and apoptotic cell death, thereby breaking the inflammatory cycle responsible for progressive renal tissue damage during sepsis.

### Histopathological results

**Fig. 8:**
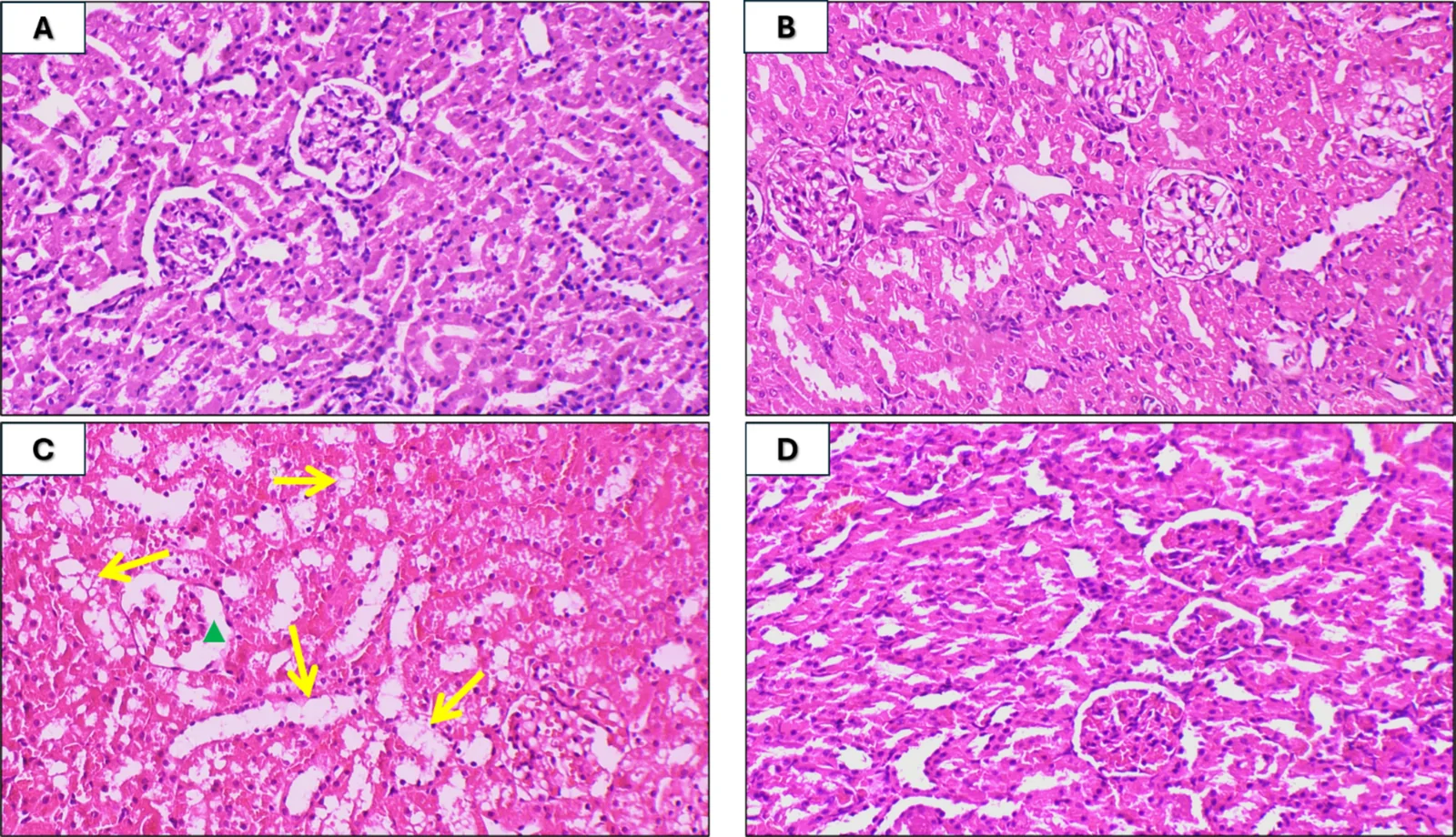
Representative photomicrographs of renal tissues (H&E staining, x 200) of the control (**A**), LCZ696 (**B**), CLP (**C**), CLP+LCZ696 (**D**) groups. Green arrowhead: dilated bowman capsule, yellow arrow: vacuolar degeneration and necrotic tubular epithelial cells.

Microscopic examination of renal tissue sections provided direct morphological evidence corroborating the biochemical and molecular findings. Kidney sections from sham-operated control animals appeared structurally intact, with well-defined renal tubules, normal glomerular architecture, and no discernible signs of cellular degeneration, inflammatory cell infiltration, or tubular disruption. In stark contrast, kidneys from CLP-treated rats exhibited severe and widespread histopathological alterations. These included marked inflammatory cell infiltration throughout the renal interstitium, prominent shedding and loss of the brush border membrane of tubular epithelial cells, extensive vacuolar degeneration of tubular cells, frank tubular epithelial cell necrosis, and conspicuous tubular dilation, as shown in Fig. 8A–D; Table 2. These structural disruptions were accompanied by significantly elevated Paller histological injury scores, confirming the severity and extent of CLP-induced renal tissue damage. The combination of tubular necrosis and dilation reflects a profound loss of tubular integrity, consistent with the impaired renal function markers observed biochemically. Importantly, administration of LCZ696 prior to CLP induction substantially ameliorated these histopathological disturbances in the CLP+LCZ696 group. Treated animals showed a remarkable reduction in inflammatory cell infiltration, near-normalization of tubular morphology, preservation of brush border integrity, and significantly lower histological injury scores compared to the untreated CLP group. These compelling morphological improvements provide strong structural evidence that LCZ696 confers meaningful renal tissue protection against the destructive consequences of sepsis-induced injury, corroborating all biochemical, oxidative, and molecular data obtained in this investigation.

**Table 2:** Histological scoring for the different groups. Each parameter (inflammation, vacuolar degeneration, tubular necrosis, and tubular dilation) was scored from 0 (none) to 3 (severe) according to the degree of inflammatory cell infiltration and renal tissue degradation and necrosis, scoring was performed by a blinded observer unaware of group allocation.

Groups	Control	LCZ696	CLP	CLP+LCZ696
**Inflammation**	0	0	2	1
**Vacuolar degeneration**	0	0	2	1
**Tubular necrosis**	0	0	3	1
**Total scoring**	0	0	7^a^	3^b^

The significance of differences was assessed using the Kruskal-Wallis test, followed by Dunn’s multiple comparison test (*n* = 6), where ^a^ indicates a significant difference relative to the control group and ^b^ indicates a significant difference relative to the CLP group.

## Discussion

AKI is a prevalent complication of sepsis that exerts a catastrophic impact on patient survival. It is estimated that approximately half of all AKI cases are attributable to sepsis, and roughly 60% of septic patients develop AKI^35^. The pathophysiological underpinnings of septic AKI are complex and remain incompletely elucidated, and similarly, the epidemiology of this condition is not yet fully characterized^36^. The mechanisms driving the onset of acute kidney injury involve a multifaceted interplay of inflammatory cascades and oxidative stress^37^.

It is also important to recognize that individuals with pre-existing cardiovascular conditions may have a compromised immune response, rendering them more susceptible to infectious complications. In the context of heart failure, diminished cardiac output and impaired circulatory function can further predispose patients to septicemia, a condition capable of triggering systemic inflammation, multi-organ dysfunction, and potentially life-threatening outcomes^38,39^. Further, it is important to note that the mortality rate among individuals with concurrent heart failure and sepsis is significantly elevated, potentially reaching up to 90%^40^.

A growing body of evidence supports the diverse beneficial health effects of LCZ696 across a variety of experimental rat disease models^41–43^. Drawing on these accumulated findings, and to the best of the authors’ knowledge, the present study constitutes the first attempt to elucidate the inhibitory effects of LCZ696 on inflammation, oxidative stress, and apoptosis in a rat model of CLP-induced septic AKI, while simultaneously examining the underlying molecular mechanisms.

Selecting an appropriate animal model is fundamental to meaningful disease research. In this study, the CLP-induced sepsis model was chosen due to its ability to closely replicate the progression of sepsis in humans, effectively reflecting the complex physiological responses, cytokine secretion dynamics, and hemodynamic perturbations observed in septic patients^44^.

The current study identified notable disruptions in renal architecture in CLP-induced rats, characterized by injured renal tubules. Concurrently, elevated MDA levels reflected a surge in reactive oxygen species, while significant reductions in SOD and GSH activity confirmed the depletion of the kidney’s antioxidant defense system relative to the sham group. Impaired renal filtration was further evidenced by elevated serum urea and creatinine concentrations in the CLP group.

Given the well-established contribution of inflammation to AKI pathogenesis, proinflammatory cytokine secretion in the kidney was examined. The findings demonstrated that CLP-induced sepsis significantly upregulated TNF-α gene expression and produced an indisputable rise in renal IL-1β and IL-18 protein levels as measured by ELISA. These observations are consistent with prior reports demonstrating elevated IL-1β and IL-6 secretion in the kidney alongside increased TNF-α, an early cytokine implicated in the pathological cascade of sepsis-induced AKI^36,45,46^. In contrast, pretreated animals with LCZ696 displayed marked improvements in renal tubular histology. Lipid peroxidation was significantly reduced, and antioxidant markers specifically SOD and GSH were notably restored compared to the model group. These findings are consistent with those reported by Mohany et al., who demonstrated that LCZ696 administration in diabetic rats reduced inflammatory markers including TNF-α, IL-1β, and IL-6. ELISA quantification of antioxidant enzyme levels in kidney homogenates also strongly supported the restoration of SOD and catalase (CAT) enzymatic activities^41^.

To further investigate the potential anti-inflammatory mechanisms underlying LCZ696 therapy in CLP-induced AKI, the expression of TLR4 was assessed by immunohistochemistry, while NLRP3 inflammasome expression was assessed by western blot analysis^47^. The TLR4/NF-κB/NLRP3 signaling pathway occupies a central role in sepsis pathogenesis^48,49^. As a constituent of the TLR family, TLR4 is deeply involved in mediating inflammatory responses during sepsis. Research has confirmed that TLR4 levels are significantly elevated in the kidneys of CLP-induced septic rats^50^, and TLR4-targeted therapies have been shown to effectively improve survival rates in such models^51^. Upon activation by bacterial LPS, TLR4 initiates downstream signaling leading to the secretion of pro-inflammatory cytokines including TNF-α, IL-1β, and IL-18^52,53^. These cytokines modulate immunological responses and promote macrophage activation, resulting in further upregulation of inflammatory gene expression^54,55^. Consistent with prior reports, the current study observed a substantial elevation in TLR4 expression in the kidneys of septic rats that was commensurate with the pronounced inflammatory response. Administration of LCZ696 markedly reversed these alterations, suggesting that modulation of the TLR4 signaling axis may be a central mechanism underlying LCZ696’s anti-inflammatory effects.

NF-κB is a family of transcriptional regulators governing a diverse array of genes associated with immune and inflammatory responses. NF-κB signaling has been found to be significantly elevated across all examined organs in septic animal models and human patients^56^. Numerous recent investigations have confirmed that NF-κB activation is linked to mortality in animal sepsis models, and its inhibition in LPS-induced sepsis significantly reduced mortality and facilitated endotoxin tolerance^57,58^. Under basal conditions, NF-κB is maintained in an inactive state through its association with IκB proteins in the cytosol. Upon cellular stress, IKK activation is initiated, driving the phosphorylation of IκB proteins. The phosphorylated IκB proteins are subsequently degraded, liberating active NF-κB, which translocates into the nucleus to initiate the transcriptional activation of target inflammatory genes.

In the present study, NF-κB gene expression was significantly activated in the renal tissues of CLP-induced septic rats relative to the sham group. Pretreatment with LCZ696 produced a substantial decrease in renal NF-κB gene expression compared to the CLP group. These findings align with those of Quagliariello et al., who demonstrated that LCZ696 confers cardioprotective effects against doxorubicin-induced cardiac injury through the downregulation of NLRP3 and NF-κB expression^59^.

Sepsis-related overactivation of the NLRP3 inflammasome may initiate a self-amplifying inflammatory cascade. The NLRP3 inflammasome is a multiprotein complex comprising caspase-1, NLRP3, and the adaptor protein ASC, and is activated by a broad spectrum of pathogen-associated molecular patterns (PAMPs) such as LPS, as well as damage-associated molecular patterns (DAMPs) including ATP, heat shock proteins, and double-stranded DNA^60^. The NLRP3 inflammasome plays a key role in immune surveillance, and its dysregulation has been implicated in numerous human pathologies. Extensive prior investigations have documented that NLRP3 inflammasome activation amplifies inflammatory responses, worsening the progression of sepsis and its associated multi-organ damage. However, pharmacological inhibition of NLRP3 activation has been shown to substantially attenuate these harmful effects^61–63^. Importantly, NF-κB activation triggered by microbial ligands such as TLR agonists or endogenous cytokines such as TNF-α is required for the upregulation of NLRP3 expression^6,64^. Consistent with this body of evidence, the current study found NLRP3 expression to be highest in the CLP-induced sepsis group as determined by western blot analysis. In the LCZ696-pretreated group, a notable reduction in NLRP3 expression was observed. This finding is consistent with the report by Shen et al. (2021), which demonstrated that sacubitril/valsartan (LCZ696) mitigated ventricular remodeling following myocardial infarction by suppressing NLRP3 inflammasome expression and the associated inflammatory response^21^. Similarly, recent research confirmed that LCZ696 effectively attenuated cyclophosphamide-induced premature ovarian failure through its inhibitory action on the NLRP3 inflammasome pathway^20^.

Apoptosis represents one of the principal mechanisms driving sepsis-induced AKI. Numerous studies have demonstrated that inhibiting caspase-3-mediated apoptosis attenuates renal injury^65,66^. Additionally, recent evidence has shown that NLRP3 inflammasome activation can trigger apoptosis alongside pyroptosis through concurrent activation of caspase-3 and caspase-1^67^. Lee et al. found that caspase-3 inhibitors confer protective effects in experimental septic mouse models^68^. The present study revealed comparable findings in renal tissue: caspase-3 protein levels were significantly elevated in sepsis-induced rats compared to sham animals, while LCZ696 pretreatment markedly attenuated renal caspase-3 levels. These results are concordant with prior work in a CLP-induced sepsis rat model demonstrating that LCZ696 significantly reduced caspase-3 expression in hepatic tissue^69^.

Several limitations of the present study should be acknowledged. First, LCZ696 was compared only against untreated CLP animals, without a valsartan-alone (or other angiotensin-receptor-blocker-alone) arm; such a comparator would help clarify whether the renoprotective effects reported here are driven predominantly by angiotensin-receptor blockade, by neprilysin inhibition, or by their combination. This design reflects the primary aim of establishing proof-of-concept for the combined ARNI approach in this model, building on separate reports that angiotensin-receptor blockade attenuates sepsis-associated renal injury; a dedicated valsartan-monotherapy comparator arm is planned for future mechanistic follow-up work. Second, the significant reduction in renal IL-18, a cytokine whose maturation strictly requires inflammasome-associated caspase-1 activity, provides indirect functional evidence that LCZ696 restrains inflammasome activation; however, we did not directly measure caspase-1 cleavage or ASC speck formation, nor did we include a pharmacological (e.g., TAK-242, MCC950) or genetic loss-of-function comparator to formally establish causality within the TLR4/NLRP3 axis. The associations reported between LCZ696 treatment and reduced TLR4/NLRP3/NF-κB signaling should therefore be regarded as correlative rather than definitive proof of a direct causal mechanism, and inhibitor- or knockout-based validation is an important direction for future work.

With respect to the TLR4/NF-κB/NLRP3 axis discussed above, high-mobility group box 1 (HMGB1), a prototypical damage-associated molecular pattern released from injured tubular cells, has recently been shown to activate this same axis and drive podocyte and glomerular injury^70^, and thioredoxin-interacting protein (TXNIP) has been shown to physically couple oxidative stress sensing to NLRP3 activation in renal and podocyte injury^71,72^; whether HMGB1 release or TXNIP-NLRP3 coupling contribute to the findings reported here was not examined in the present study and represents an important avenue for future mechanistic dissection, alongside the gut-microbiota-dependent mechanisms noted below.

Additional biomarkers of tubular injury, such as kidney injury molecule-1 and neutrophil gelatinase-associated lipocalin, urinary albumin-to-creatinine ratio, and Masson trichrome staining for early fibrotic change, would further strengthen future assessments of renal injury and recovery in this model; these were not obtained in the present 24-hour acute study, which precedes the typical window for fibrotic remodeling, but would be valuable in longer-term follow-up protocols. Likewise, emerging evidence indicates that the gut microbiota and its metabolites are important regulators of the septic inflammatory response and of macrophage metabolic reprogramming, with interventions such as time-restricted feeding and microbiota-derived indole metabolites conferring protection against sepsis-induced organ injury in preclinical models^73,74^. Whether such microbiota-dependent mechanisms intersect with the renoprotective actions of LCZ696 observed here remains an open question that merits dedicated future investigation. Finally, this study was conducted exclusively in male rats, and LCZ696 was administered as a 6-day pretreatment beginning before CLP induction; while this design isolates the drug’s protective potential under controlled experimental conditions, it does not model the more clinically realistic scenario in which treatment begins after sepsis onset. Future studies incorporating female cohorts and post-insult dosing regimens will be important to confirm the translational relevance of these findings.

## Conclusion

In summary, the findings of the present investigation demonstrate that a six-day pretreatment regimen with LCZ696, administered prior to CLP surgery, conferred substantial protection against sepsis-induced acute kidney injury in rats. These renoprotective effects appear to be mediated through modulation of the TLR4/NF-κB/NLRP3 inflammasome signaling pathway, resulting in the attenuation of oxidative stress markers, restoration of antioxidant enzymatic activity, and meaningful reduction in pro-IL-1β, pro-IL-18, and TNF-α levels. Based on these observations, LCZ696 pretreatment may represent a novel and promising therapeutic approach given its demonstrated capacity to preserve renal function and directly protect renal tissue from the deleterious consequences of sepsis-induced injury.

## Supplementary Information

Below is the link to the electronic supplementary material.

## Data Availability

All data produced or examined during this investigation are incorporated in this published article.
