# Correction: Sacubitril/valsartan attenuates renal injury caused by cecal ligation and puncture via TLR4/NFκB/NLRP3 inhibition and reduced oxidative stress and apoptosis in rats

**DOI:** 10.1038/s41598-026-68366-0

**Published:** 2026-09-02

**Authors:** Reham H. Mohyeldin, Mahmoud Abdelnaser, Remon Roshdy Rofaeil, Mina Ezzat Attya, Niera M. S. Soliman, Michael Samuel Ayad, Ehab E. Sharata

**Affiliations:** 1 Department of Pharmacology & Toxicology, Faculty of Pharmacy, Deraya University, Minia, 61111 Egypt; 2 Department of Biochemistry, Faculty of Pharmacy, Deraya University, Minia, 61111 Egypt; 3 Department of Medical Pharmacology, Faculty of Medicine, Minia University, 61519 Minia, Egypt; 4 Department of Pathology, Faculty of Medicine, Minia University, Minia, 61519 Egypt; 5 Department of Medical Physiology, Faculty of Medicine, Minia University, Minia, 61519 Egypt; 6 Vascular Surgery Department, Faculty of Medicine, Minia University, Minia, 61519 Egypt

Correction to: *Scientific Reports* https://doi.org/10.1038/s41598-026-65493-6, published online 11 August 2026

The original version of this Article contained an error in the spelling of the author Niera M. S. Soliman which was incorrectly given as Niera M. S. Niera.

The original Article has been corrected.

