# Supplementary material for: Sacubitril/valsartan attenuates renal injury caused by cecal ligation and puncture via TLR4/NFκB/NLRP3 inhibition and reduced oxidative stress and apoptosis in rats
Source: Sci Rep. 2026 Aug 11;16:24919. doi: 10.1038/s41598-026-65493-6 (PMC13462680; doi:10.1038/s41598-026-65493-6)
Supplement: Supplementary file 1 [file 41598_2026_65493_MOESM1_ESM.pdf]

Figure 1A. Serum urea (mg/dL), n = 6 per group

Group	Animal 1	Animal 2	Animal 3	Animal 4	Animal 5	Animal 6	Mean ± SD
**Sham**	15.67	16.88	18.70	14.00	17.06	19.54	16.97 ± 2.01
**LCZ696**	17.22	20.90	13.57	16.69	17.32	15.05	16.79 ± 2.48
**CLP**	38.65	35.98	36.05	40.06	37.77	45.06	38.93 ± 3.38
**CLP+LCZ696**	22.67	27.87	30.66	28.06	27.47	28.63	27.56 ± 2.65

Figure 1B. Serum creatinine (mg/dL), n = 6 per group

Group	Animal 1	Animal 2	Animal 3	Animal 4	Animal 5	Animal 6	Mean ± SD
**Sham**	0.83	0.87	0.56	0.71	0.67	0.73	0.73 ± 0.11
**LCZ696**	0.57	0.69	0.50	0.74	0.80	0.71	0.67 ± 0.11
**CLP**	1.96	2.34	1.95	2.08	2.17	1.88	2.06 ± 0.17
**CLP+LCZ696**	1.20	1.09	1.40	1.32	1.34	1.18	1.25 ± 0.12

Figure 2A. Renal MDA (nmol/g tissue), n = 6 per group

Group	Animal 1	Animal 2	Animal 3	Animal 4	Animal 5	Animal 6	Mean ± SD
**Sham**	2.92	1.97	1.95	3.22	3.97	3.83	2.98 ± 0.88
**LCZ696**	3.77	2.83	1.86	3.36	2.22	3.99	3.01 ± 0.85
**CLP**	7.30	6.68	7.76	8.16	7.22	8.50	7.60 ± 0.67
**CLP+LCZ696**	3.79	4.63	4.26	5.53	3.33	4.97	4.42 ± 0.80

Figure 2B. Renal GSH (mg/g tissue), n = 6 per group

Group	Animal 1	Animal 2	Animal 3	Animal 4	Animal 5	Animal 6	Mean ± SD
**Sham**	3.77	3.55	3.69	3.36	3.96	2.54	3.48 ± 0.50
**LCZ696**	4.06	3.23	3.06	2.98	3.60	3.20	3.35 ± 0.41
**CLP**	1.05	1.33	0.99	1.87	1.18	1.51	1.32 ± 0.33
**CLP+LCZ696**	2.30	2.18	2.04	2.16	2.75	2.38	2.30 ± 0.25

Figure 2C. Renal SOD (U/g tissue), n = 6 per group

Group	Animal 1	Animal 2	Animal 3	Animal 4	Animal 5	Animal 6	Mean ± SD
**Sham**	3.90	3.75	3.55	3.98	3.44	3.82	3.74 ± 0.21
**LCZ696**	3.94	3.47	3.51	3.66	3.57	3.88	3.67 ± 0.20

Group	Animal 1	Animal 2	Animal 3	Animal 4	Animal 5	Animal 6	Mean ± SD
**CLP**	2.24	1.95	2.45	2.31	2.30	2.45	2.28 ± 0.18
**CLP+LCZ696**	3.21	3.00	2.68	3.11	3.22	3.01	3.04 ± 0.20

Figure 3A. Renal IL-1β (pg/mg protein), n = 6 per group

Group	Animal 1	Animal 2	Animal 3	Animal 4	Animal 5	Animal 6	Mean ± SD
**Sham**	380.92	340.39	370.38	350.78	290.46	360.14	348.85 ± 31.95
**LCZ696**	360.42	320.96	300.90	340.55	310.82	370.70	334.06 ± 27.90
**CLP**	720.44	620.85	650.52	710.06	700.08	770.47	695.40 ± 53.05
**CLP+LCZ696**	460.06	440.48	450.37	470.30	380.03	460.60	443.64 ± 32.77

Figure 3B. Renal IL-18 (pg/mg protein), n = 6 per group

Group	Animal 1	Animal 2	Animal 3	Animal 4	Animal 5	Animal 6	Mean ± SD
**Sham**	170.23	180.67	210.09	160.14	170.94	200.47	182.09 ± 19.34
**LCZ696**	200.44	170.06	180.69	160.13	190.73	210.45	185.42 ± 18.86
**CLP**	370.72	380.94	350.04	420.06	390.93	420.35	388.84 ± 27.81
**CLP+LCZ696**	230.73	260.15	240.64	280.27	220.12	260.77	248.78 ± 22.26

Figure 4. Renal caspase-3 (ng/mg protein), n = 6 per group

Group	Animal 1	Animal 2	Animal 3	Animal 4	Animal 5	Animal 6	Mean ± SD
**Sham**	13.34	16.13	12.69	13.56	14.09	12.36	13.70 ± 1.34
**LCZ696**	14.32	12.78	12.49	15.84	12.13	12.97	13.42 ± 1.40
**CLP**	36.89	28.57	28.64	27.39	29.84	32.75	30.68 ± 3.55
**CLP+LCZ696**	18.25	20.26	22.84	19.36	16.51	18.64	19.31 ± 2.13

Figure 5A. Renal NF-κB mRNA (fold change vs sham), n = 6 per group

Group	Animal 1	Animal 2	Animal 3	Animal 4	Animal 5	Animal 6	Mean ± SD
**Sham**	1.02	0.97	0.90	0.89	1.00	1.00	0.96 ± 0.06
**LCZ696**	1.07	0.88	1.11	0.86	1.10	1.09	1.02 ± 0.12
**CLP**	4.23	5.03	4.21	3.82	4.79	4.84	4.49 ± 0.47
**CLP+LCZ696**	3.05	2.67	2.54	2.37	2.92	2.95	2.75 ± 0.27

Figure 5B. Renal TNF-α mRNA (fold change vs sham), n = 6 per group

Group	Animal 1	Animal 2	Animal 3	Animal 4	Animal 5	Animal 6	Mean ± SD
**Sham**	1.12	0.94	0.89	0.81	1.04	1.10	0.98 ± 0.12
**LCZ696**	1.09	0.91	0.87	0.79	1.02	1.07	0.96 ± 0.12
**CLP**	3.63	4.17	3.93	3.52	4.08	4.11	3.91 ± 0.27
**CLP+LCZ696**	2.41	2.18	2.75	2.04	2.59	2.73	2.45 ± 0.29

Figure 6B. TLR4 IHC positivity (% positive area), n = 3 per group

Group	Animal 1	Animal 2	Animal 3	Mean ± SD
**Sham**	9.55	11.77	12.00	11.11 ± 1.35
**LCZ696**	12.00	10.70	12.90	11.87 ± 1.11
**CLP**	41.44	38.00	36.00	38.48 ± 2.75
**CLP+LCZ696**	24.00	27.00	28.00	26.33 ± 2.08

Figure 7B. NLRP3 protein (western blot) (fold change vs sham), n = 3 per group

Group	Animal 1	Animal 2	Animal 3	Mean ± SD
**Sham**	1.00	0.94	1.09	1.01 ± 0.08
**LCZ696**	1.01	1.11	0.95	1.02 ± 0.08
**CLP**	5.77	6.25	5.40	5.81 ± 0.43
**CLP+LCZ696**	2.65	2.40	2.85	2.63 ± 0.23
