# Supplementary material for: Sacubitril/valsartan attenuates renal injury caused by cecal ligation and puncture via TLR4/NFκB/NLRP3 inhibition and reduced oxidative stress and apoptosis in rats
Source: Sci Rep. 2026 Aug 11;16:24919. doi: 10.1038/s41598-026-65493-6 (PMC13462680; doi:10.1038/s41598-026-65493-6)

Repeat 1

NLRP3

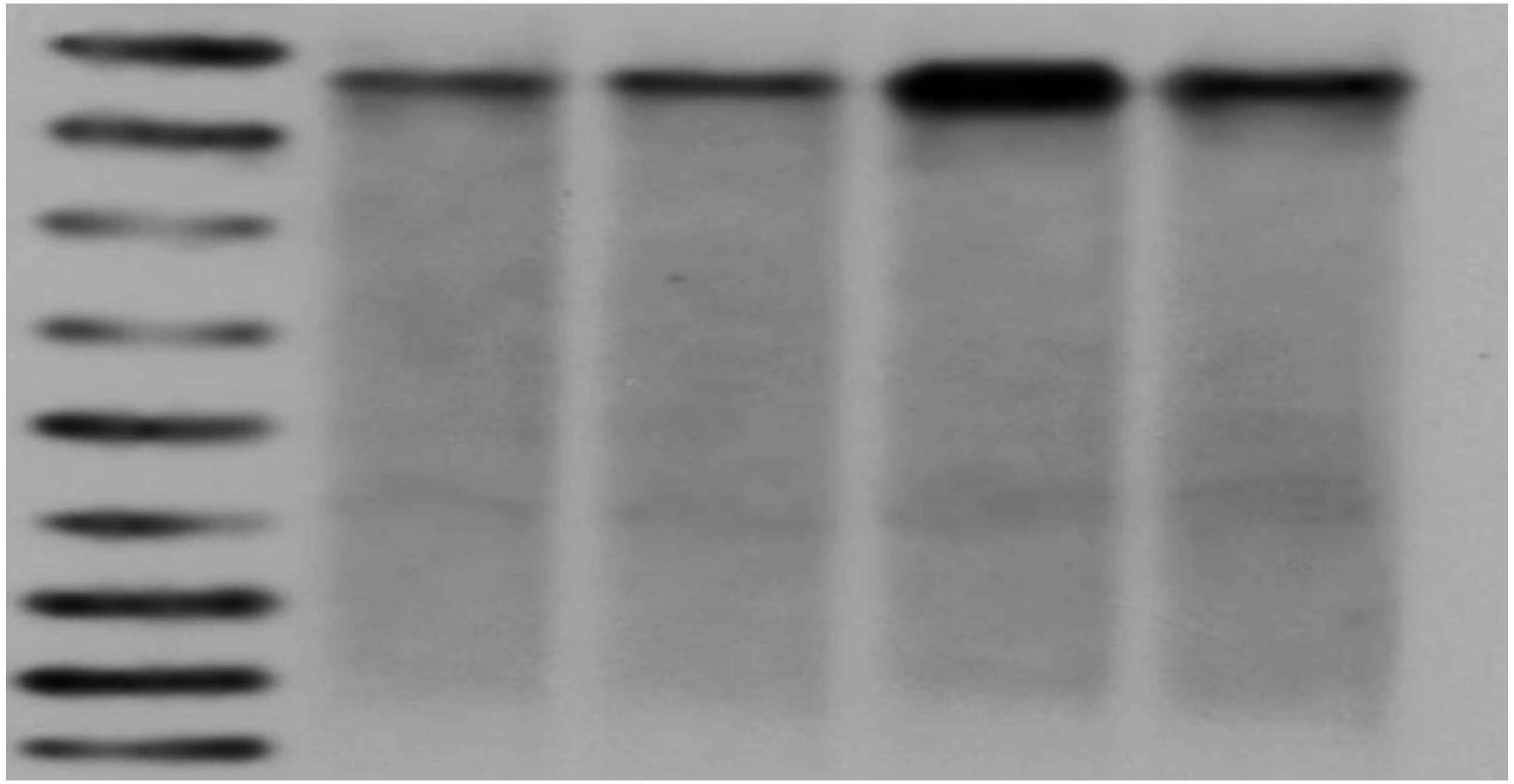

NLRP3

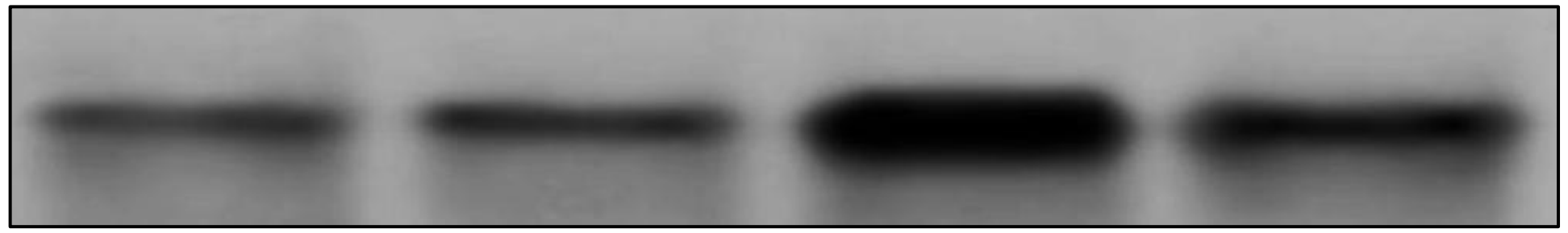

118 KDa

Repeat 1

B-actin

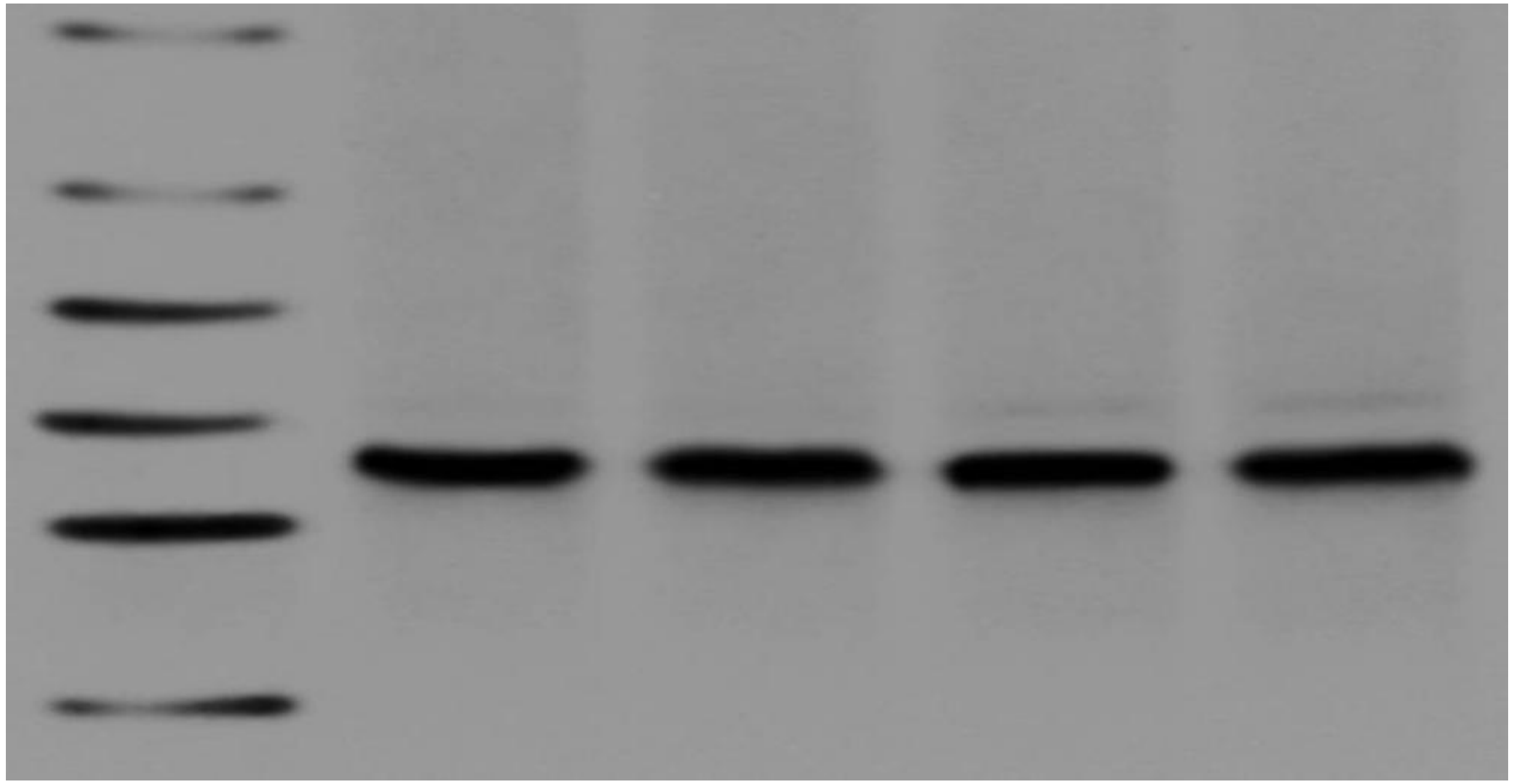

B-actin

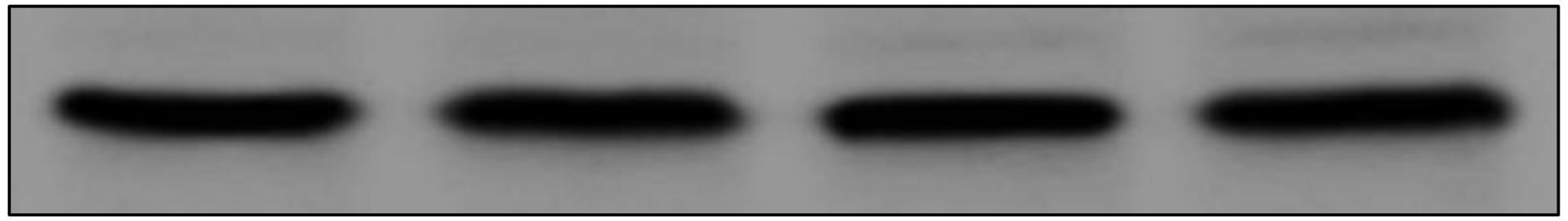

43 KDa

Repeat 2

NLRP3

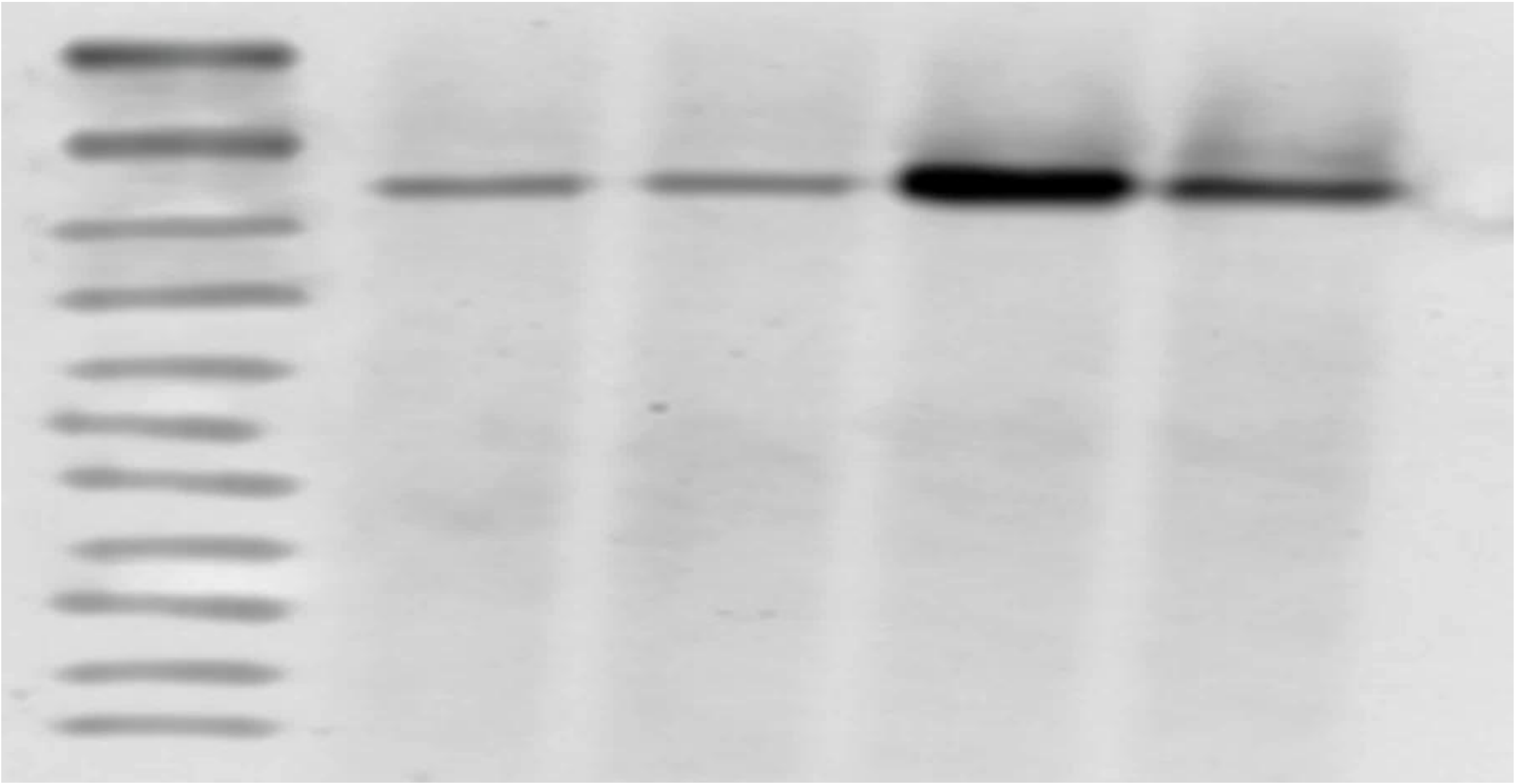

NLRP3

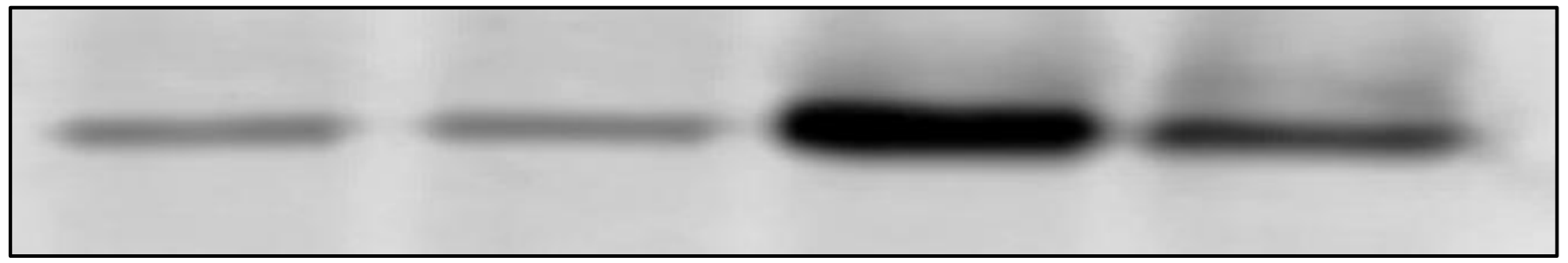

118 KDa

Repeat 2

B-actin

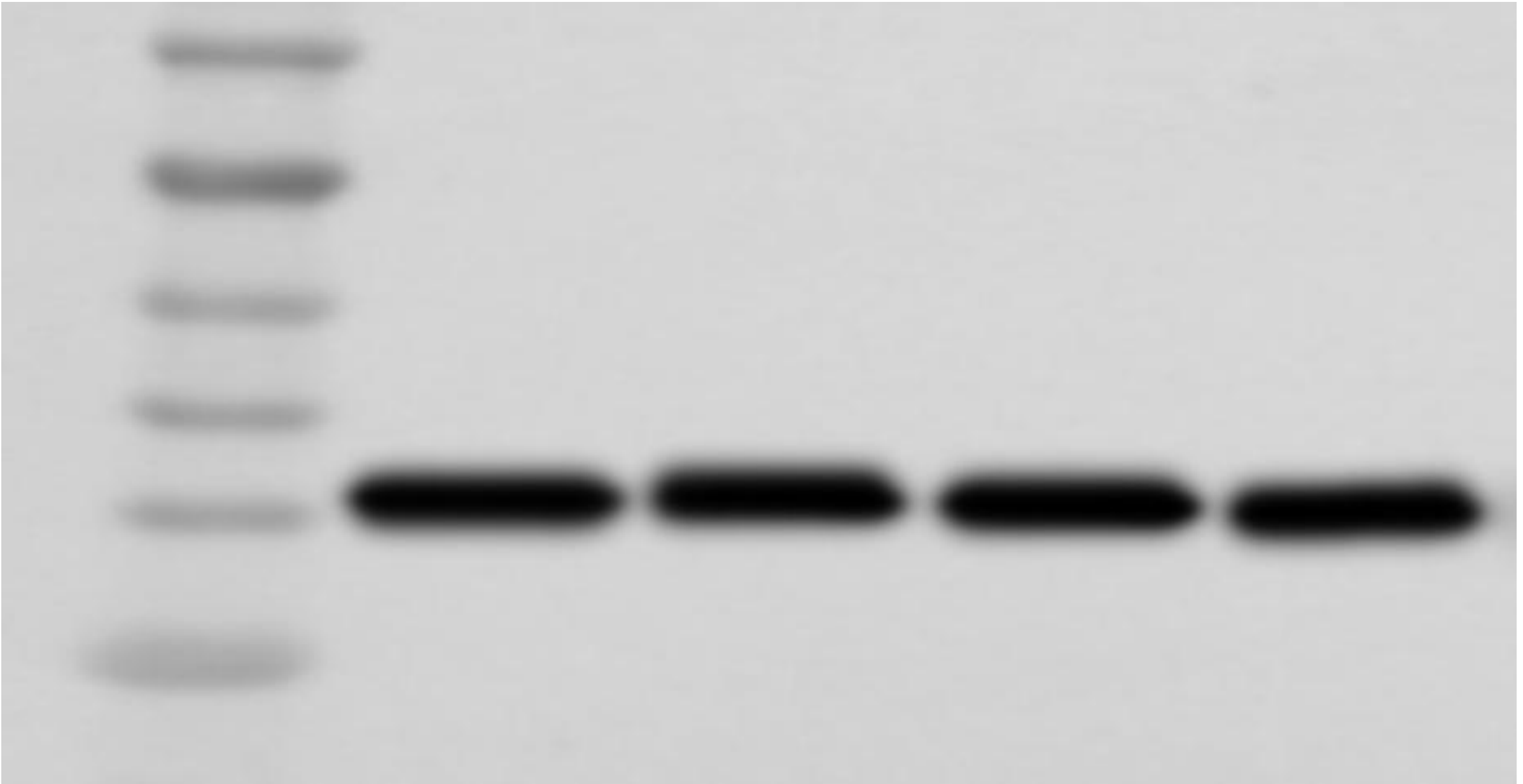

B-actin

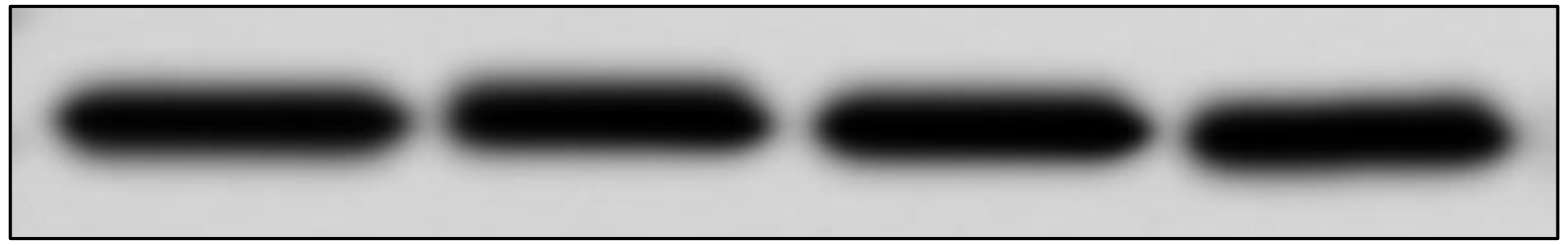

43 KDa

Repeat 3

NLRP3

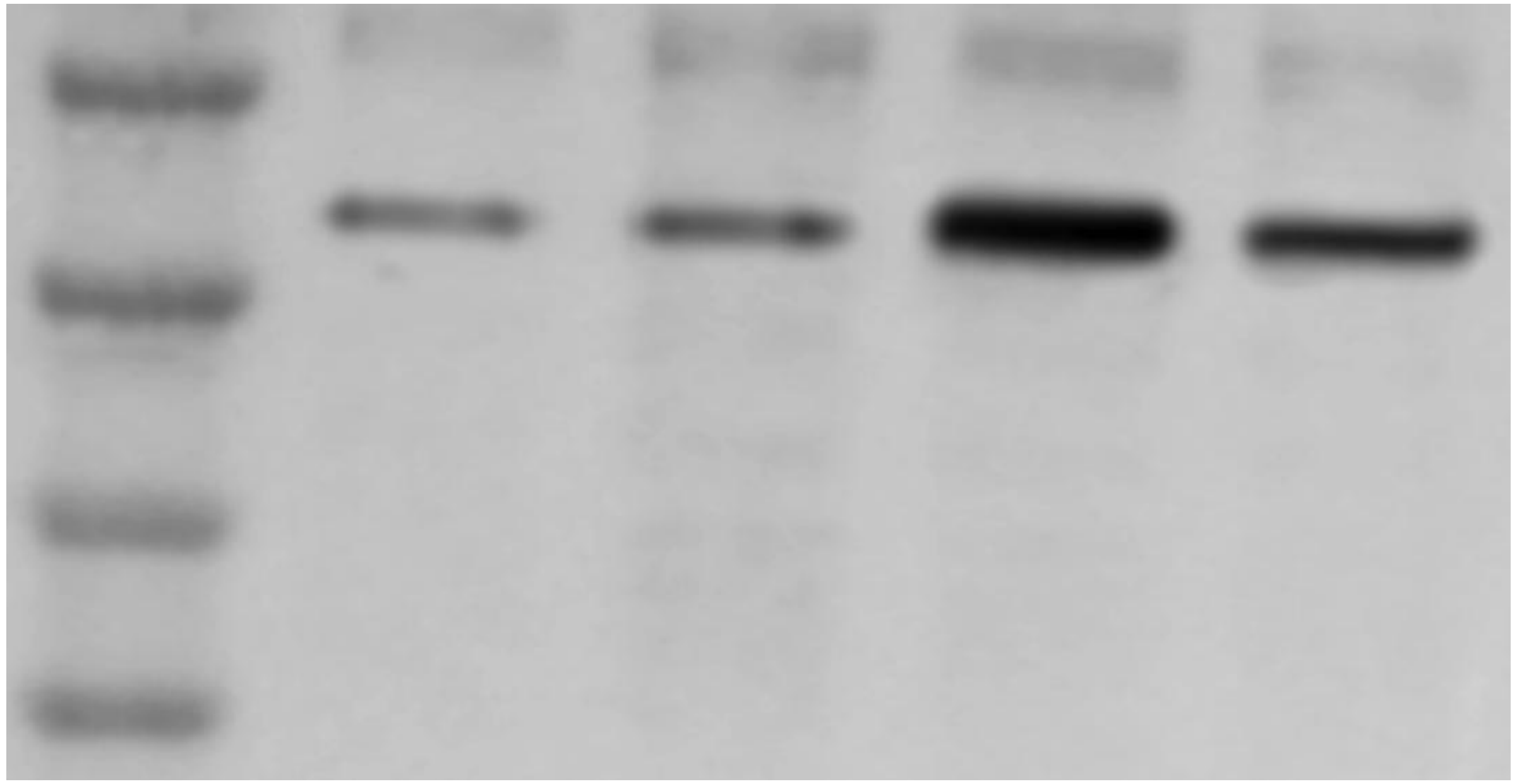

NLRP3

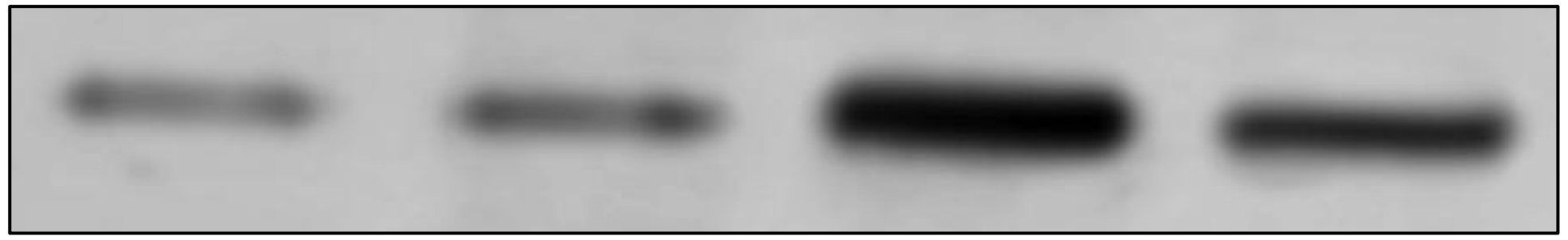

118 KDa

Repeat 3

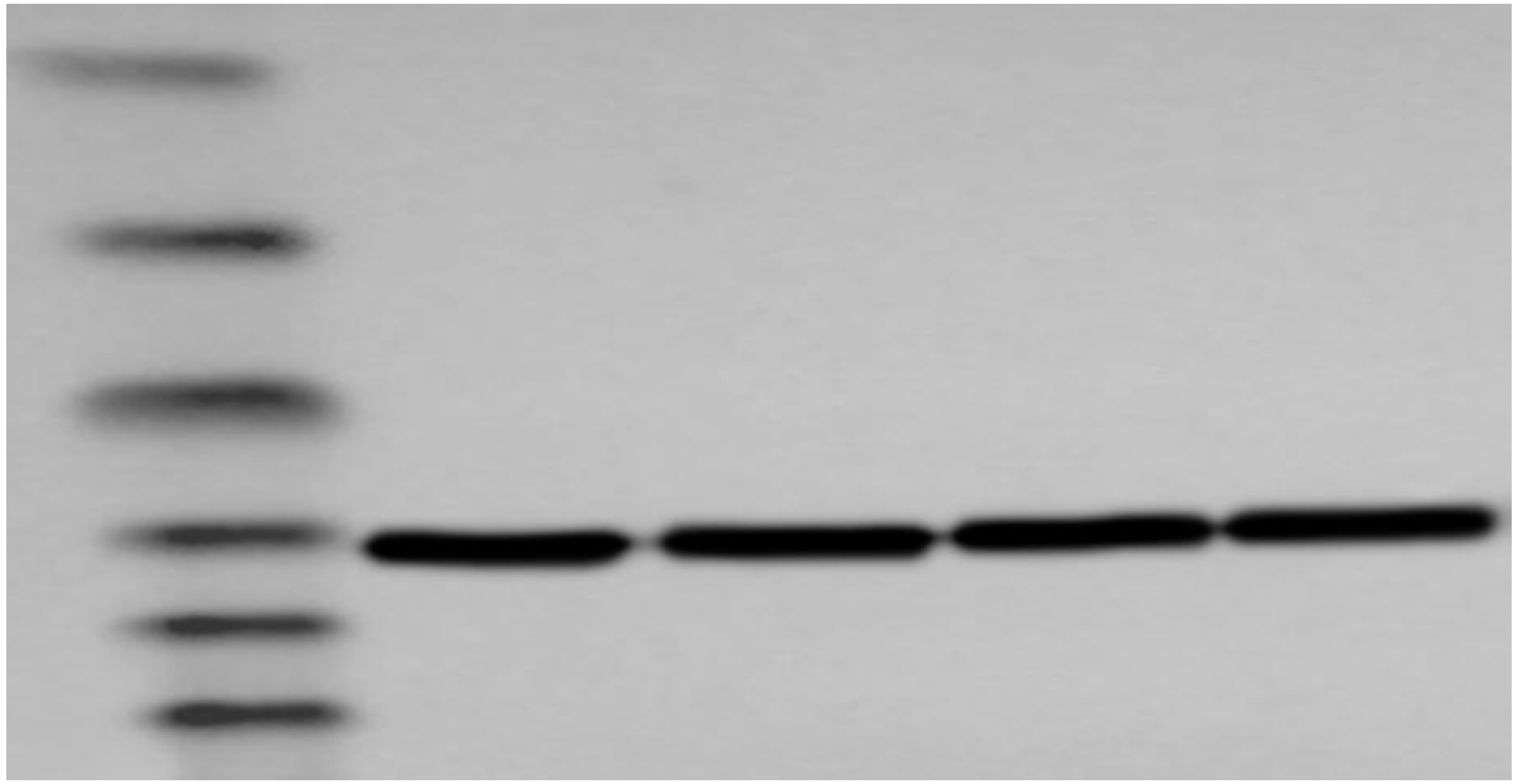

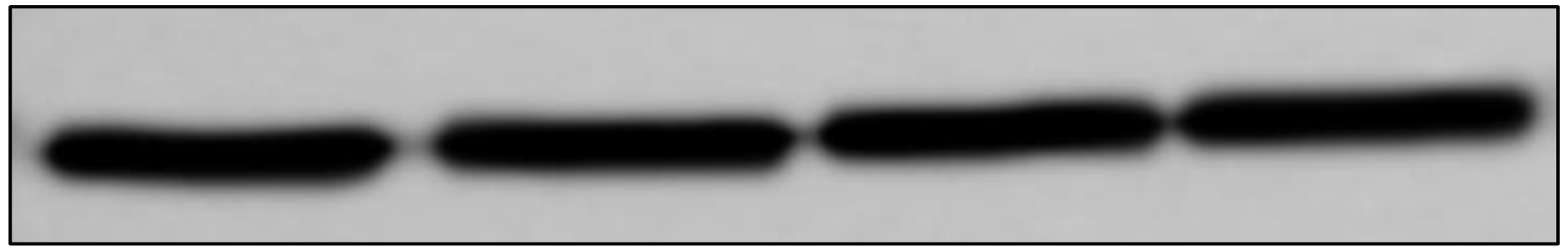

Supplement: Supplementary file 2 [file 41598_2026_65493_MOESM2_ESM.pdf]
